# Sphingosine kinase 2 regulates adipocyte browning and whole-body metabolism

**DOI:** 10.1126/sciadv.aec7066

**Published:** 2026-08-21

**Authors:** Ryan D. R. Brown, Cynthia Weigel, Yadu Vijayan, Katarzyna M. Tyc, Christiane Carter, Christopher D. Green, Sarah Spiegel

**Affiliations:** ^1^ Department of Cellular, Molecular and Genetic Medicine, Virginia Commonwealth University, Richmond, VA 23298, USA.; ^2^ Department of Biostatistics, School of Public Health, Virginia Commonwealth University, Richmond, VA 23298, USA.; ^3^ Bioinformatics Shared Resource, Massey Comprehensive Cancer Center, Virginia Commonwealth University, Richmond, VA 23298, USA.

## Abstract

Obesity increases circulating levels of the bioactive sphingolipid metabolite sphingosine-1-phosphate (S1P). Here, we identify adipocyte-expressed sphingosine kinase 2 (SPHK2), an enzyme that produces S1P, as a regulator of adipose tissue browning. Mice with adipocyte-specific deletion of *Sphk2* were protected from Western diet–induced obesity concomitantly with elevated energy expenditure and browning of subcutaneous fat. *Sphk2* deletion also enhanced expression of *Ucp1* and key thermogenic genes, resulting in reduced adiposity and hepatic steatosis, improved glucose tolerance, and insulin sensitivity. Mechanistically, obesogenic diet promoted nuclear localization of SPHK2 in adipocytes, where it cooperated with transcription factors to suppress the thermogenic transcriptome. Adipocyte SPHK2 contributes to homeostatic circulating S1P levels and drive the pathogenic rise of S1P in response to obesogenic diet. Our findings reveal a distinct role of adipocyte SPHK2 in regulation of the thermogenic program important for whole-body metabolic homeostasis and energy expenditure, highlighting SPHK2 as a potential therapeutic target for treatment of metabolic disorders.

## INTRODUCTION

Obesity is a global health crisis associated with an increased risk for type 2 diabetes, liver and cardiovascular diseases, and cancer (*1*). Obesity arises from an imbalance between energy intake and expenditure and is influenced by genetic, epigenetic, behavioral, and environmental factors, such as overconsumption of fat and sugar. Adipose tissue is an endocrine organ essential in coordinating whole-body energy metabolism, by storing excess energy as large, unilocular lipid droplets in white adipocytes or facilitating thermogenesis and energy dissipation in brown and beige adipocytes (*2*). Additionally, it produces secreted factors known as adipokines, including leptin and adiponectin. Once the storage capacity of adipose tissue is exceeded as in obesity, excess lipids are deposited in other organs, including the liver and muscle, contributing to the development of insulin resistance and metabolic dysfunction–associated steatotic liver disease (MASLD) (*2*, *3*). This relationship between adipose tissue and the liver underscores the systemic impact of obesity on metabolic health (*3*). Promoting adipose remodeling to reduce lipid accumulation has been suggested to be a potential therapeutic strategy for obesity and its metabolic comorbidities (*3*).

In addition to the canonical accumulation of neutral lipids, such as triglycerides (TGs) and cholesterol, overnutrition also increases the bioactive sphingolipid metabolites ceramide and sphingosine-1-phosphate (S1P) in the circulation and tissues (*4*–*6*), and their dysregulation correlates with obesity, insulin resistance, steatosis, and the development of murine and human metabolic dysfunction–associated steatohepatitis (MASH) (*7*–*11*). In obese mice, adipose-specific deletion of serine palmitoyltransferase (SPTLC2) or dihydroceramide desaturase 1 (DES1), key enzymes initiating ceramide biosynthesis, or of ceramide synthase 6 (CERS6) that produces C16:0 ceramide specifically in brown adipocytes, alter adipose tissue remodeling, induce subcutaneous adipose browning, boost mitochondrial activity, and improve insulin sensitivity (*4*, *5*, *9*, *12*). In contrast, promoting ceramide accumulation by deletion of acid ceramidase (*Asah1*) from mature thermogenic adipocytes led to decreased energy expenditure and worsened insulin resistance and diet-induced obesity (*13*). These studies also emphasize the importance of ceramides in diet-induced dysfunction of thermogenic adipocytes (*13*). Yet, much less is known of the functions of its further metabolite S1P and the sphingosine kinases, SPHK1 and SPHK2, that produce it in adipose tissue.

Most studies on S1P acting as a ligand for its receptors S1PR1-3 expressed in adipocytes were carried out using 3T3-L1 cells, and only a few validated their biological functions in vivo. It was suggested that activation of S1PR1 and S1PR3 inhibit, while S1PR2 stimulates, preadipocyte differentiation (*14*). However, during the later stages of adipogenesis, expression of S1PR1-3 decreases, and exogenous S1P does not restore adipogenesis, suggesting that the S1PRs may not be sufficient for this process (*15*). Nevertheless, *S1pr2^−/−^* mice fed a high-fat diet (HFD) had reduced adipocyte hypertrophy, decreased adipose tissues inflammation, and improved insulin sensitivity (*14*). Whereas insulin resistance and glucose intolerance were exacerbated in HFD-fed *S1pr3^−/−^* mice (*16*), suggesting opposing roles for these S1PRs.

Global loss of *Sphk1* ameliorates steatosis and adipocyte proinflammatory responses (*17*). In contrast, TG accumulation in large lipid droplets was increased in brown adipose tissue (BAT) of these *Sphk1*^−/−^ mice due to reduced lipophagy (*18*). Moreover, adipocyte-specific *Sphk1* deletion exacerbated adipocyte hypertrophy and glucose intolerance with impaired lipolysis (*19*). Similarly, tissue-specific opposing functions of the other isoenzyme SPHK2 in metabolic diseases have been reported (*20*–*25*). Global deletion of *Sphk2* protected mice from obesity and insulin resistance induced by Western diet (WD) (*20*, *21*), likely by increasing lipolysis of adipose tissue (*20*) or by suppressing lipotoxicity of pancreatic β cells (*22*). Moreover, inhibition of SPHK2 with ABC294640 suppressed HFD-induced obesity (*26*), and K145 ameliorated hepatic lipid accumulation in ob/ob or db/db mice (*27*). However, while WD-induced obesity and insulin resistance are reduced in *Sphk*2^−/−^ mice (*20*, *21*), yet mice with hepatocyte-specific *Sphk2* deletion still develop severe insulin resistance and glucose intolerance (*25*), suggesting that the beneficial effects of *Sphk2* deletion may not reside within the hepatocyte compartment. Because adipose tissue plays a crucial role in metabolic homeostasis regulation, and abundant evidence indicates that adipose tissue dysfunction links obesity to insulin resistance (*2*, *28*), herein we set out to investigate the intrinsic role of adipocyte SPHK2 in these processes using mice specifically lacking *Sphk2* in mature adipocytes, referred to as *Sphk2*^∆Adipo^. These mice were protected from WD-induced obesity, metabolic impairment, and development of hepatic steatosis. Deletion of *Sphk2* in adipose tissue increased expression of thermogenic genes leading to the browning of subcutaneous fat, reduced adiposity, and improved glucose tolerance, insulin sensitivity, and β-adrenergic responsiveness. Mechanistically, we found that, upon WD feeding, SPHK2 was localized to the nucleus where it promotes repression of thermogenic genes in white adipose tissue (WAT). Adipocyte SPHK2 was also responsible for maintaining homeostatic circulating S1P levels and the pathogenic rise of S1P in response to diet. Our findings uncover a previously unrecognized and distinct role of adipose SPHK2 in regulating the thermogenic program important for metabolic homeostasis and energy expenditure. Moreover, we suggest that SPHK2 regulates the pathogenic rise of circulating S1P associated with obesity and related comorbidities.

## RESULTS

### Adipocyte-specific deletion of *Sphk2* attenuates WD-induced obesity and accumulation of S1P

**Fig. 1.**
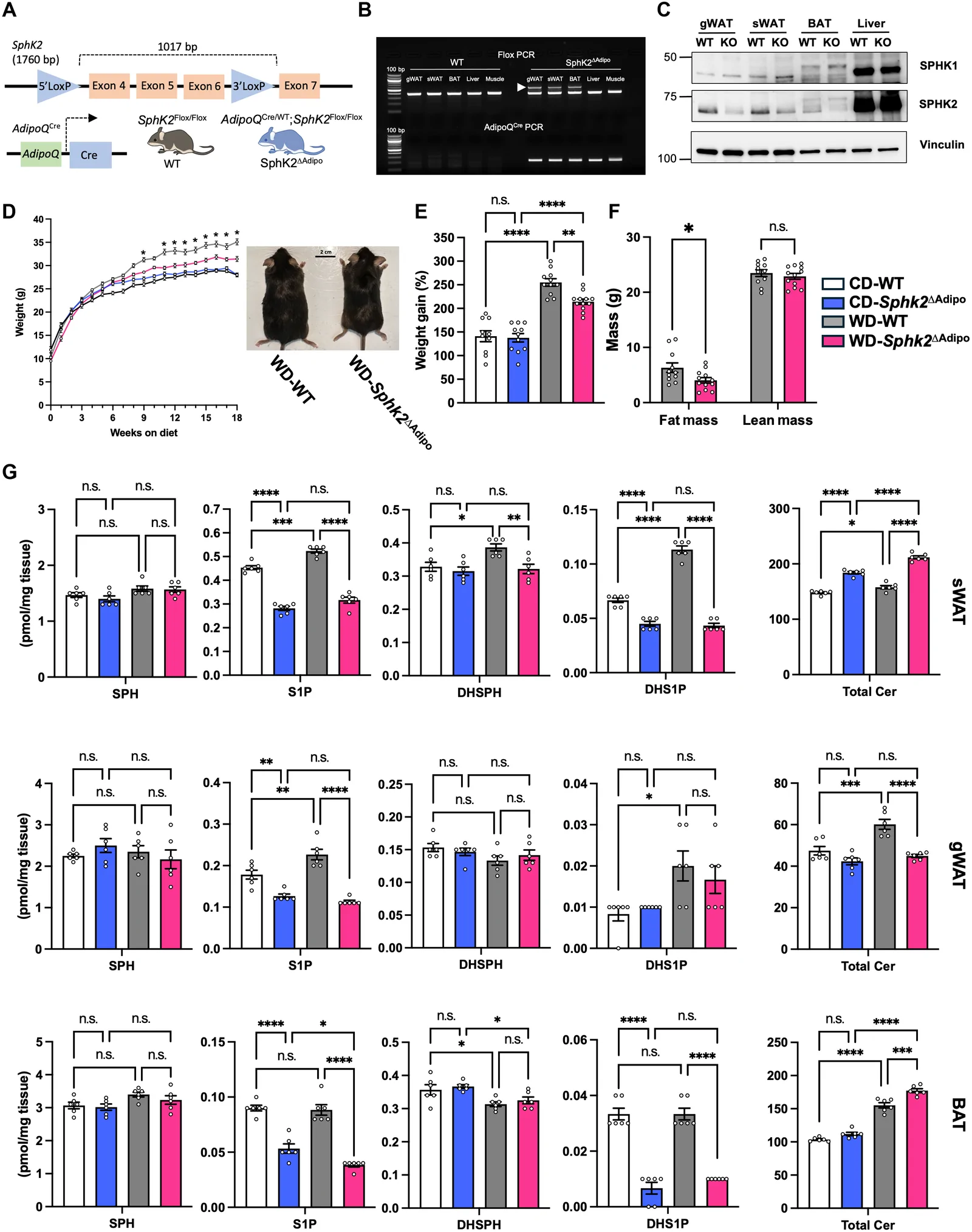
Mice lacking SPHK2 in adipocyte are resistant to diet-induced obesity and S1P accumulation. (**A**) Schematic depicting the generation of *Sphk*2^∆Adipo^ mice by Cre-LoxP deletion of *Sphk2* exons 4 to 6 in *Adipoq*-expressing adipocytes. (**B**) Representative DNA agarose gel showing Cre-recombinase expression (200 base pairs) and WT *Sphk2* flox/flox (391 base pairs) and *Sphk2* deletion (arrowhead; 560 base pairs) specifically in adipose from transgenic mice. (**C**) Representative Western blots of SPHK1 and SPHK2 in gWAT, sWAT, BAT, and liver from male wild-type (WT) and *Sphk2*^∆Adipo^ (KO) mice (*N* = 3). (D to G) WT and *Sphk2*^∆Adipo^ male mice were fed chow or WD for 18 weeks as indicated. (**D**) Body weights. Lines represent CD-WT (black), CD-*Sphk2*^ΔAdipo^ (blue), WD-WT (gray), and WD-*Sphk2*^ΔAdipo^ (pink). (**E**) Weight gain. (**F**) Body composition (*N* = 10 to 13). (**G**) Sphingolipids were extracted from gWAT, sWAT, and BAT, and the levels of sphingosine (SPH), dihydrosphingosine (DHSPH), sphingosine-1-phosphate (S1P), dihydrosphingosine-1-phosphate (DHS1P), and total ceramide were determined by liquid chromatography–electrospray ionization–tandem mass spectrometry (LC-ESI-MS/MS) (*N* = 6). Data are presented as means ± SEM. (D) Two-way analysis and (E to G) one-way analysis of variance (ANOVA) test followed by Šidák’s multiple comparisons test. bp, base pairs; n.s., not significant.

We generated mice harboring adipocyte-specific deletion of *Sphk2*, termed *Sphk2*^∆Adipo^ by crossing mice containing a floxed *Sphk2* allele with those carrying a transgenic Cre recombinase driven by the *Adipoq* promoter (Fig. 1A) to delete *Sphk2* at a developmental stage after adipocyte lineage commitment (*29*). The selective excision of the targeted exons of *Sphk2* in adipose tissue depots was confirmed by polymerase chain reaction (PCR) analysis of genomic DNA (Fig. 1B). Specific deletion of SPHK2 protein was evident in visceral gonadal WAT (gWAT), inguinal subcutaneous WAT (sWAT), and axial BAT, without affecting its level in the liver (Fig. 1C and fig. S1A). The remaining SPHK2 expression is likely due to immune and stromal cells also present in the adipose. Furthermore, depletion of SPHK2 in adipose tissue had no compensatory effect on levels of SPHK1 (Fig. 1C and fig. S1A).

To explore the involvement of adipocyte-specific depletion of *Sphk2* in the pathophysiology of diet-induced obesity, *Sphk2*^∆Adipo^ and wild-type (WT) littermate controls of both sexes were fed standard chow diet or a high-fat, high-cholesterol, and carbohydrate-rich diet with high-glucose and high-fructose sugar water. This recapitulated a typical WD and mimicked key metabolic, transcriptomic, and histologic changes associated with obesity and development of MASLD/MASH in humans (*30*). As metabolic diseases are sexually dimorphic (*31*), the effects of adipose *Sphk2* deletion were evaluated separately in male and female mice. As expected, WT mice fed WD had significantly greater weight gains compared with chow-fed littermates, with greater increases in males than in females (Fig. 1, D and E, and fig. S1B). At baseline, body weight did not differ significantly between genotypes in either sex (Fig. 1E and fig. S1B). A small difference (∼2.5 g) in one male cohort was within the normal range and not statistically significant. Moreover, although *Adipoq*-Cre can target subsets of bone marrow stromal cells (*32*), robust recombination was detected in adipose tissue (Fig. 1B), whereas only a minimal signal was observed in bone marrow as expected. Diet-induced weight gain was attenuated in both male and female *Sphk2*^∆Adipo^ mice (Fig. 1, D and E, and fig. S1B). Accordingly, analysis of body composition revealed that obesogenic-fed *Sphk2*^∆Adipo^ male and female mice had significantly reduced fat mass (Fig. 1F and fig. S1C).

Next, it was of interest to examine levels of bioactive sphingolipid metabolites, S1P and ceramide, that have been correlated with obesity in humans (*4*–*6*). Consistent with previous studies (*4*, *5*, *19*, *33*), WD feeding increased the levels of S1P measured by liquid chromatography–electrospray ionization–tandem mass spectrometry (LC-ESI-MS/MS) in gWAT and sWAT of male and female mice (Fig. 1G and fig. S1D). Although ceramide was also increased in all of these tissues from WD fed males, in females, however, increased ceramide was noted only in the BAT (fig. S1D). Adipocyte-specific SPHK2 deletion markedly reduced levels of S1P and dihydroS1P (DHS1P) in these adipose depots in both sexes (Fig. 1G and fig. S1D), suggesting that SPHK2 is the predominant isoform responsible for the diet-induced increase in S1P and DHS1P, rather than SPHK1. The effects of *Sphk2* deletion on ceramide levels varied in fat tissues and genders (Fig. 1G and fig. S1D). However, irrespective of gender or diet, SPHK2 ablation reduced ceramide levels in gWAT (Fig. 1G and fig. S1D). Moreover, *Sphk2* ablation had no major effects on levels of sphingosine or dihydrosphingosine (Fig. 1G and fig. S1D). These results suggest that SPHK2 in adipose primarily affects the levels of its products S1P and DHS1P in these tissues.

### Adipocyte-selective ablation of *Sphk2* in obese mice alters bioenergetics and improves adipose remodeling

**Fig. 2.**
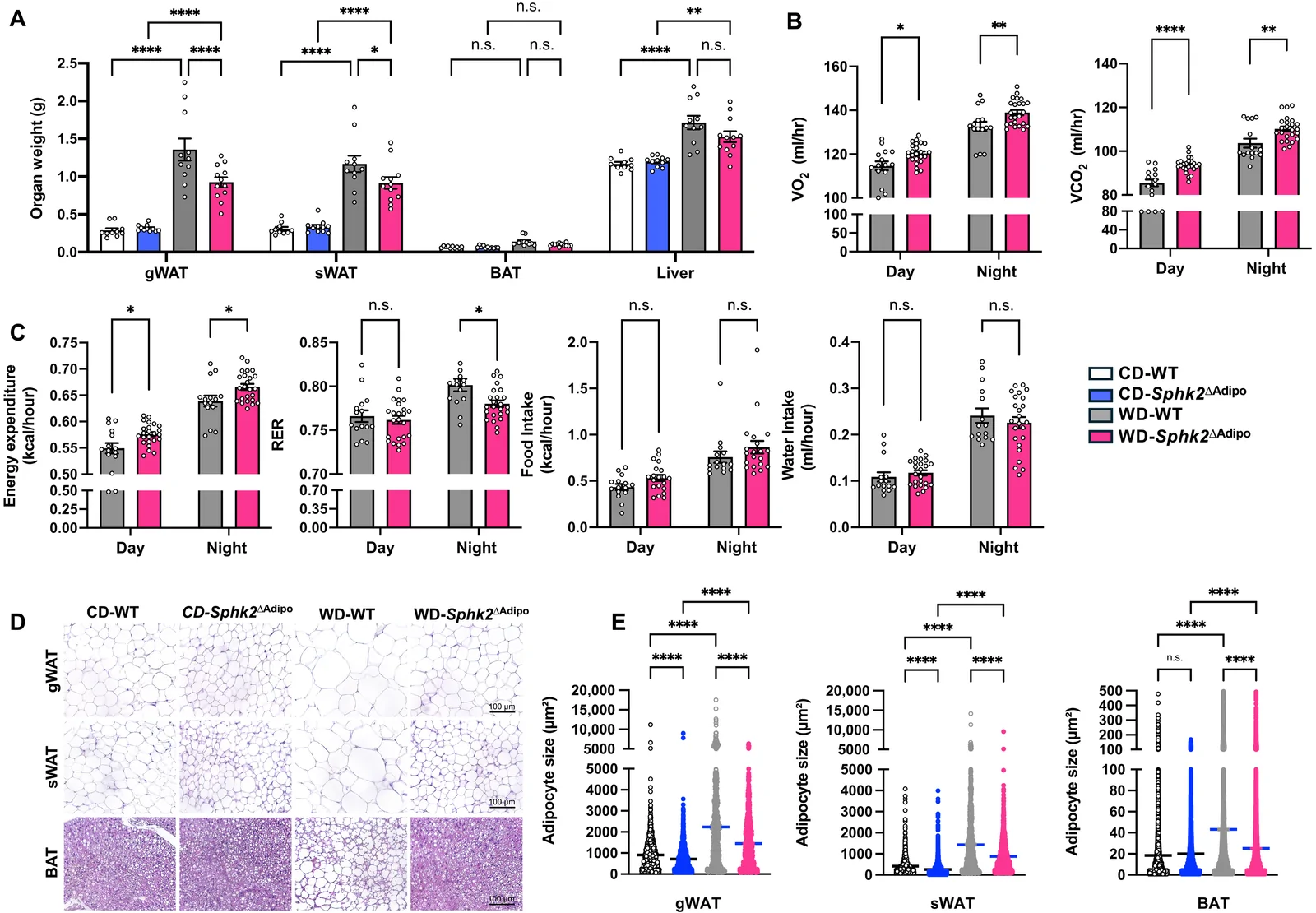
Adipocyte-specific ablation of SPHK2 in obese male mice increases energy expenditure and reduces adipocyte hypertrophy. (A to E) Male *Sphk2*^∆Adipo^ mice and WT littermates were fed chow or WD for 18 weeks. (**A**) Organ weights were measured (*N* = 10 to 12). (**B** and **C**) After 16 weeks of WD feeding, mice were individually housed in metabolic chambers for 48 hours to acclimate; water consumption, food intake, energy expenditure, O_2_ consumption (VO_2_), CO_2_ production (VCO_2_), and respiratory exchange ratio (RER) were measured during the subsequent 4 days; and data are displayed as average per day (*N* = 16 to 24). (**D**) Representative images of hematoxylin and eosin–stained gWAT, sWAT, and BAT. (**E**) Adipocyte sizes were determined for each of the depots (*N* = 6). Data are presented as means ± SEM. (A) Two-way ANOVA test followed by Tukey’s multiple comparisons test. (B and C) Two-way ANOVA test followed by Šidák’s multiple comparisons test. (E) One-way ANOVA test followed by Tukey’s multiple comparisons test. n.s., not significant.

It was of interest to further explore why *Sphk2*^∆Adipo^ mice are resilient to WD-induced weight gain in greater detail. Further analyses of both sexes indicated reduced gWAT and sWAT adipose mass, with trending reductions in BAT mass and liver weights (Fig. 2A and fig. S2A). Notably, the site of adipose tissue reduction in visceral or subcutaneous depots has profound impacts on metabolic health (*2*). However, minimal effects on weight gain (Fig. 1E and fig. S1B) and organ weights (Fig. 2A and fig. S2A) were observed in chow-fed *Sphk2*^∆Adipo^ mice compared with those in WT counterparts. Obesogenic diet-fed *Sphk2*^∆Adipo^ male and female mice displayed increased levels of oxygen consumption (VO_2_), carbon dioxide production (VCO_2_), and whole-body energy expenditure, independent of changes in food and water intake compared with WT mice (Fig. 2, B and C, and fig. S2, B and C). Moreover, *Sphk2*^∆Adipo^ mice also had a reduced respiratory exchange ratio (RER) (Fig. 2C and fig. S2C), which suggests greater utilization of lipid β-oxidation over carbohydrates as primary fuel sources. The unhealthy increase in fat by WD is largely due to adipocyte hypertrophy (increase in cell size), rather than hyperplasia (associated with de novo adipogenesis), and is closely linked to dyslipidemia and systemic insulin resistance (*34*). Histological analysis showed that the obesogenic diet markedly increased the size of gonadal, subcutaneous, and brown, adipocytes in both sexes (Fig. 2, D and E, and fig. S2, D and E). Cross-sectional areas of adipocytes from WD-fed *Sphk2*^∆Adipo^ male mice were 35, 39, and 52% lower in sWAT, eWAT, and BAT, respectively, compared with those of adipocytes from WD-fed WT male mice (Fig. 2E). Similar reductions were found in WD-fed *Sphk2*^∆Adipo^ female mice (fig. S2E). Small yet significant reductions in adipocyte sizes in sWAT and gWAT were also noted in chow-fed *Sphk2*^∆Adipo^ mice. Collectively, these data indicate that adipocyte-specific SPHK2 deletion is responsible, at least, in part, for the antiobesogenic effects observed in global *Sphk2*^−/−^ mice (*20*, *21*).

### Adipocyte SPHK2 ablation protects both sexes from WD-induced metabolic dysfunction and governs the circulating sphingolipidome

**Fig. 3.**
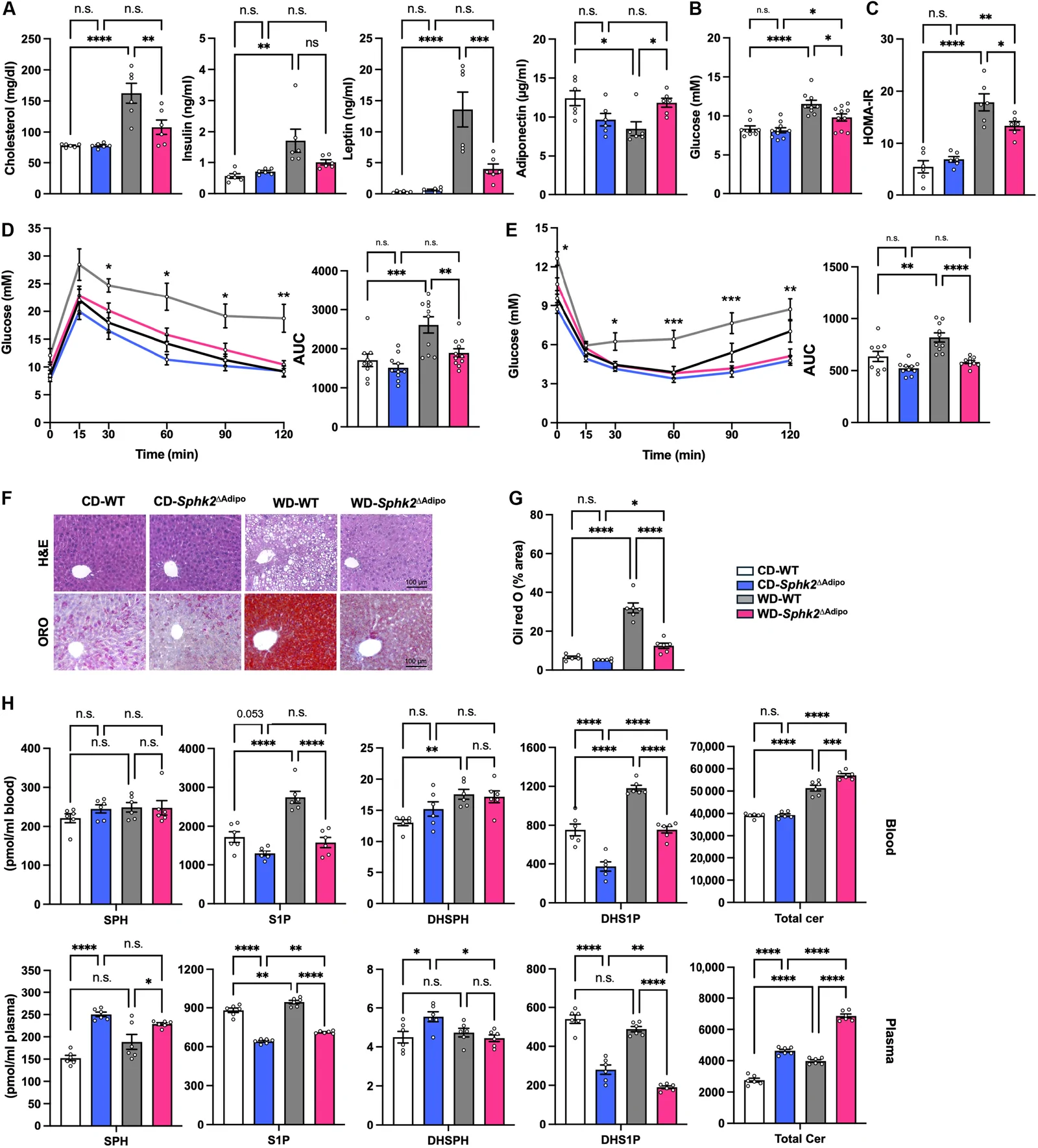
Adipocyte-specific *Sphk2* deletion improves glucose homeostasis, mitigates hepatic steatosis, and regulates circulating levels of S1P in obese mice. (A to H) *Sphk2*^∆Adipo^ and WT littermate male mice were fed chow or WD for 15 (D and E) or 18 (A to C and F to H) weeks. (**A**) Plasma concentrations of cholesterol, insulin, leptin, and adiponectin (*N* = 6). (**B**) Blood glucose levels (*N* = 8 to 11). (**C**) Homeostatic model assessment of insulin resistance (HOMA-IR) (*N* = 6). (**D**) Glucose tolerance and area under the curve (AUC). (**E**) Insulin tolerance and AUC (*N* = 8 to 11). (D and E) Lines represent CD-WT (black), CD-*Sphk2*^ΔAdipo^ (blue), WD-WT (gray), and WD-*Sphk2*^ΔAdipo^ (pink). (**F**) Representative images of hematoxylin and eosin (H&E)– and oil red O (ORO)–stained liver sections after 18 weeks chow or WD feeding. (**G**) Oil red O staining was quantified as percent staining per total tissue surface area (*N* = 6). (**H**) Sphingolipids were extracted from blood and plasma, and the levels of sphingosine (SPH), dihydrosphingosine (DHSPH), sphingosine-1-phosphate (S1P), dihydrosphingosine-1-phosphate (DHS1P), and total ceramide (cer) were determined by LC-ESI-MS/MS (*N* = 6). Data are presented as means ± SEM. (A to C, G, and H) One-way ANOVA test followed by Tukey’s multiple comparisons test. (D and E) Unpaired two-tailed *t* test at each time point comparing WD-WT to WD-*Sphk2*^∆Adipo^, and AUC is one-way ANOVA test followed by Tukey’s multiple comparisons test. n.s., not significant.

Adipose remodeling has a major impact on the release of lipids, hormones, and metabolites into the circulation to communicate with other metabolically active organs, such as the liver, for maintaining overall metabolic health (*2*, *35*). The decreased adiposity in *Sphk2*^∆Adipo^ mice fed WD compared with that in the *Sphk2*^*f*l/fl^ control littermates was associated with a diminution of circulating cholesterol, insulin, and leptin levels with concurrent elevation in circulating adiponectin, an insulin-sensitizing adipokine (Fig. 3A), suggesting improved adipose function.

Both male and female *Sphk2*^∆Adipo^ mice maintained on the obesogenic diet also had decreased WD-induced hyperglycemia, improved glucose tolerance, and enhanced glucose disposal during an insulin tolerance test (Fig. 3, B and E, and fig. S3, A to C), as also shown by a reduction in the homeostatic model assessment for insulin resistance (HOMA-IR) (Fig. 3C). Marginal changes in liver morphology were apparent between the genotypes on chow diet (Fig. 3F and fig. S3D). Whereas, as expected, obesogenic diet feeding of WT mice led to ectopic hepatic lipid accumulation and development of fatty liver, hepatic steatosis did not develop in both male and female *Sphk2*^∆Adipo^ mice (Fig. 3F and fig. S3D), which was confirmed by reduced oil red O staining of neutral lipids (Fig. 3, F and G, and fig. S3, F and E). Collectively, these results suggest that adipocyte-specific loss of SPHK2 is associated with improved systemic metabolic homeostasis in the obese state.

Prior studies have shown that the adipose sphingolipids, ceramide, and S1P increase in obesity (*4*, *5*, *19*, *33*) and correlate with insulin resistance and hepatic steatosis (*7*–*11*). Moreover, when ceramides are reduced in adipose of obese mice due to *Asah1* overexpression in white adipose or conditional ablation of *Sptlc2* in adipocytes, there was also a corresponding lowering of ceramides in plasma (*36*) or serum (*13*). Thus, we asked whether reduction of adipose S1P affects circulating sphingolipids levels. We also observed increased blood S1P and DHS1P levels in response to obesogenic diet in both sexes, whereas pathogenic ceramide was especially increased in males (Fig. 3H and fig. S3F). Blood levels of S1P and DHS1P were reduced in these *Sphk2*^∆Adipo^ mice (Fig. 3H and fig. S3F). This is in sharp contrast to elevation of blood S1P and DHS1P in global or hepatocyte-specific *Sphk2* knockout mice (*37*), probably due to reduced clearance by the liver (*38*).

As erythrocytes were suggested to be the main producers of S1P in the blood stream and apolipoprotein M in high-density lipoproteins and albumin transport S1P in plasma (*39*) to exclude the contribution of erythrocytes, we also measured sphingolipid levels in plasma. S1P and DHS1P were notably reduced in *Sphk2*^∆Adipo^ mice, regardless of sex or diet (Fig. 3H and fig. S3F). This indicated that adipocyte SPHK2 is, at least, partially responsible for production of homeostatic circulating S1P and contributes to its pathogenic increase in obesity. Adipose-specific deletion of SPHK2 also led to increased level of its substrate sphingosine in plasma (Fig. 3H and fig. S3F). Moreover, even in the presence of substantial systemic metabolic benefits of adipocyte-specific deletion of SPHK2 in obese mice, ceramide levels were further increased in plasma and blood (Fig. 3H and fig. S3F). Collectively, these findings indicate that adipocyte SPHK2-produced S1P contributes to diet-induced adipocyte dysfunction and to the circulating pool and the pathogenic rise in S1P.

### Adipocyte-specific deletion of *Sphk2* up-regulates the thermogenic transcriptome

**Fig. 4.**
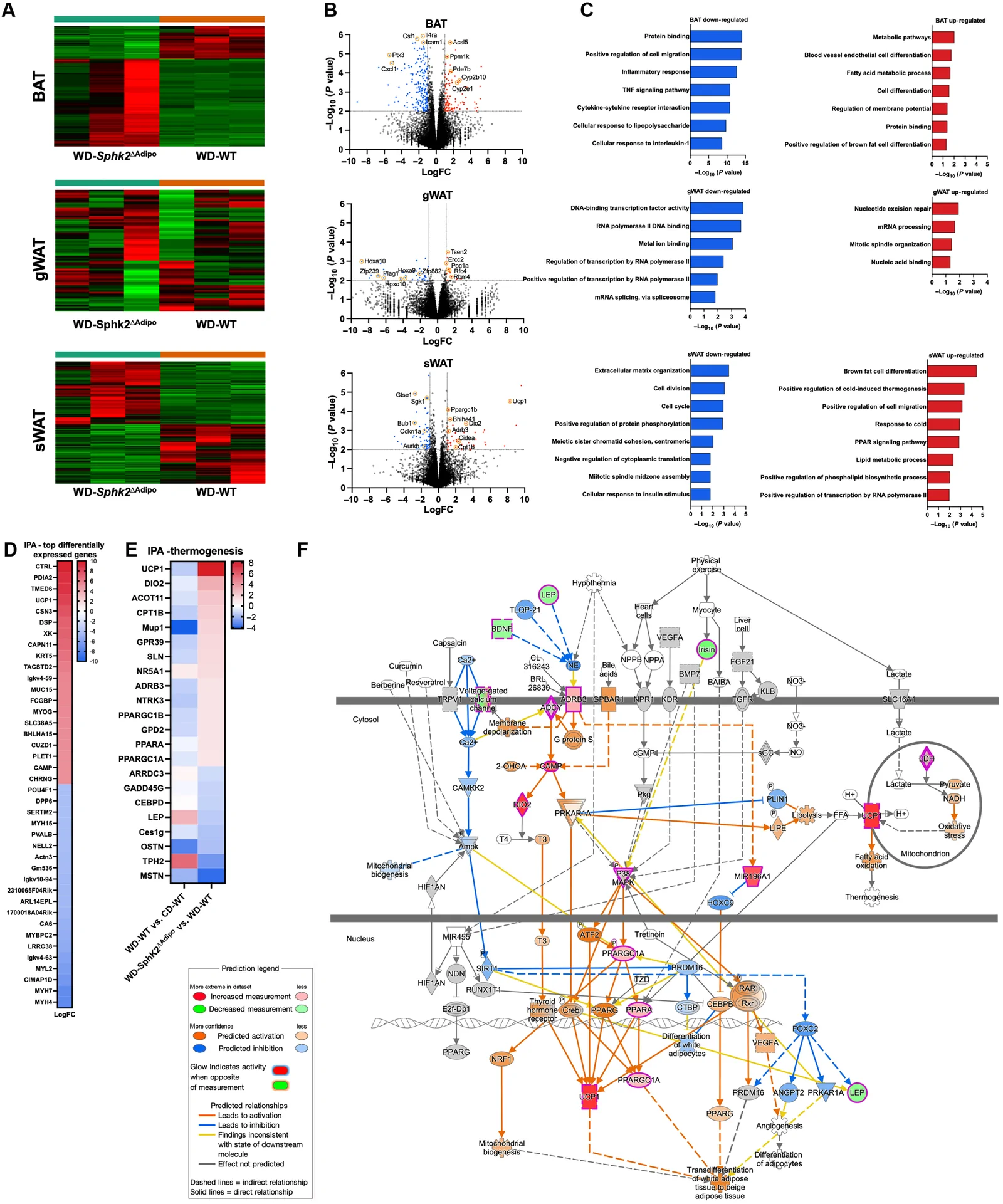
Effect of adipocyte-specific deletion of *Sphk2* on the adipose tissue transcriptome. (A to F) WT and *Sphk2*^∆Adipo^ male littermates were fed chow or WD for 18 weeks. (**A**) Heatmap expression matrix of differentially expressed genes in BAT, gWAT, and sWAT from the indicated mice. (**B**) Gene signatures of BAT, gWAT, and sWAT from WD-fed *Sphk2*^∆Adipo^ mice compared with those from WD-fed WT visualized as volcano plots. Red and blue dots represent significantly up-regulated or down-regulated genes, respectively. Note that several relevant significantly up-regulated or down-regulated genes are also encircled in orange (*N* = 3) (*P* ≤ 0.01, logFC of ±1). (**C**) Gene Ontology (GO) and Kyoto Encyclopedia of Genes and Genomes (KEGG) biological processes pathway enrichment analysis of significantly differentially expressed genes up-regulated or down-regulated in BAT, gWAT, and sWAT from WD-fed *Sphk2*^∆Adipo^ mice compared with those from WD-fed WT (*N* = 3) (*P* < 0.01, logFC of ±1). TNF, tumor necrosis factor. (**D** to **F**) Transcriptomic changes analyzed by IPA in sWAT from WD-fed *Sphk2*^∆Adipo^ mice compared with those from WD-fed WT (N = 3) (*P* ≤ 0.05, logFC of ±1). (D) The top 20 up-regulated and down-regulated genes. (E) Heatmap of the differentially expressed genes involved in thermogenesis or thermoregulation. (F) Graphical summary of the canonical WAT browning pathway. The genes shown in red are up-regulated and those that are green are down-regulated. The intensity of the colors indicates that the degree each gene was up- or down-regulated. A solid line represents a direct interaction between the two gene products, and a dotted line represents an indirect interaction.

As a first step to understand how SPHK2 so profoundly effects adipocyte remodeling and their metabolic functions, which are predominantly under transcriptional control (*2*, *40*), we performed RNA sequencing (RNA-seq) of the different adipose tissues to examine the effects of SPHK2 deletion in an unbiased approach. As expected, WD markedly altered differential gene expression in adipose tissue leading to 422 unique genes significantly changed in gWAT [286 up-regulated and 136 down-regulated; absolute log fold change (logFC) of ±1, *P* ≤ 0.01], 765 differentially expressed in sWAT (484 up-regulated and 281 down-regulated) and 789 in BAT (530 up-regulated and 259 down-regulated) (fig. S4A). These gene changes, likely as suggested by others, resulted in pathway enrichment of lipogenesis and lipid accumulation in gWAT and sWAT (*2*, *40*, *41*) and led to increased inflammatory responses in BAT (fig. S4B) (*42*). In particular, it was of interest to evaluate changes induced by *Sphk2* deletion in the transcriptomes of these obese adipose tissues. Global gene expression analysis identified 67 unique genes significantly changed in gWAT (22 up-regulated and 45 down-regulated; absolute logFC of ±1, *P* ≤ 0.01), whereas 108 genes in sWAT (51 up-regulated and 57 down-regulated) and 292 genes in BAT (98 up-regulated and 194 down-regulated) were differentially expressed in WD-fed *Sphk2*^∆Adipo^ mice compared with those in WT counterparts (Fig. 4A). Kyoto Encyclopedia of Genes and Genomes (KEGG) and Gene Ontology (GO) pathway enrichment analyses of the transcriptional profiles were subsequently used to identify potential processes affected by *Sphk2* deletion. Most of the down-regulated genes in BAT from WD-fed *Sphk2*^∆Adipo^ mice depicted in the volcano plot (Fig. 4B), including *Cxcl1*, *Csf1*, *IL4ra*, *Icam1*, and *Ptx3*, were important for inflammation, immune cell migration, and survival functions (Fig. 4C). Notably, *Cyp2e1*, which regulates lipid catabolism and *Acsl5*, responsible for fatty acyl–coenzyme A production and fueling β-oxidation, appeared in the top-most up-regulated BAT genes (Fig. 4C). Furthermore, Gene Set Enrichment Analysis (GSEA) for molecular functions and biological processes identified many inflammatory pathways such as inflammatory response, tumor necrosis factor signaling pathway and cytokine activity that appeared in the top 20 most down-regulated pathways (Fig. 4C). Conversely, although very few pathways were significantly up-regulated in the BAT of *Sphk2*^∆Adipo^ mice fed WD, the list included metabolic pathways, alongside fatty acid metabolic processes and positive regulation of brown fat cell differentiation. These interconnected pathways enable the BAT to effectively burn energy and generate heat through thermogenesis (Fig. 4C).

We found relatively few differentially expressed genes in the gWAT from WD-fed *Sphk2*^∆Adipo^ compared with those in controls (Fig. 4B), and those were not directly relatable to adipocyte function. Moreover, pathway analysis of the gWAT revealed several up-regulated and down-regulated pathways related to transcription and DNA repair; however, none of these were known to be canonically associated with adipocyte function (Fig. 4C).

Many of the genes that were down-regulated by *Sphk2* deletion in sWAT, including *Cdkn1a*, *Bub1*, and *Gtse1*, were related to cell cycle regulation (Fig. 4, B and C). Intriguingly, on the other hand, there was marked up-regulation of *Ucp1*, which uncouples mitochondrial respiration from adenosine 5′-triphosphate (ATP) synthesis and is central to regulating energy balance and thermogenesis (*43*, *44*), alongside other key adipocyte browning-associated genes, such as *Ppargc1b*, a transcription factor coactivator that enhances expression of mitochondrial metabolism and thermogenesis genes (*45*), and *Dio2* that converts the inactive thyroid hormone T4 (thyroxine) into the active form T3 (triiodothyronine) important for adaptive thermogenesis (*46*). Furthermore, many other thermogenic related genes were also significantly increased in sWAT from WD-fed Sphk2^∆Adipo^ mice: *Cidea*, which further drives the transcription of *Ucp1* for thermogenesis (*47*); *Cpt1b* is essential for fatty acid oxidation, which fuels thermogenesis; and *Adrb3* the receptor that triggers lipolytic and thermogenic cellular machinery (Fig. 4B) (*48*). Collectively, these markedly enhanced genes equate to highly up-regulated pathways related to adipose browning, namely, diet-induced thermogenesis, positive regulation of cold-induced thermogenesis, and brown fat cell differentiation (Fig. 4C).

Intrigued by these findings and to gain deeper insight, we further analyzed the transcriptomic changes in the sWAT using ingenuity pathway analysis (IPA). Once again, *Ucp1* appeared at the top of the most differentially up-regulated genes (Fig. 4D). Curating our dataset with the integrated knowledge base platform indicated that the most significantly up-regulated biological processes were thermogenesis and thermoregulation (Fig. 4E). In agreement with previous reports (*49*), many of the genes responsible for thermogenesis and thermoregulation were significantly down-regulated in the sWAT from WD-fed WT mice compared with those from chow-fed controls, whereas most of these were conversely increased in WD-fed *Sphk2*^∆Adipo^ mice (Fig. 4E). The algorithm also indicated that genes involved in thermogenesis and thermoregulation pathways intersect with those implicated in beiging, also known as browning of WAT, as illustrated in Fig. 4F highlighting central genes, pathways, and predictions of potential regulatory networks involved in this process.

### In subcutaneous adipose, SPHK2 regulates expression of thermogenesis and lipolysis genes

BAT contains a high number of mitochondria expressing uncoupling protein 1 (UCP1), which allows it to generate heat by uncoupling the proton gradient in the mitochondria instead of synthesizing ATP (*43*, *44*, *50*). Beige adipocytes present within sWAT in both rodents and humans share thermogenic properties with BAT by conditionally expressing *Ucp1* in response to cold exposure, hormones, or signals resulting in β-adrenergic receptor activation (*51*). The quantity and activity of these thermogenic UCP1 expressing adipocytes alter systemic energy metabolism, increasing substrate oxidation and energy expenditure while decreasing adiposity (*52*). As we observed an increase in energy expenditure in WD-fed *Sphk2*^∆Adipo^ mice without corresponding changes in food intake (Fig. 2), we questioned whether *Sphk2* deletion in adipocytes promoted the browning/beiging of sWAT and/or increased BAT activity.

**Fig. 5.**
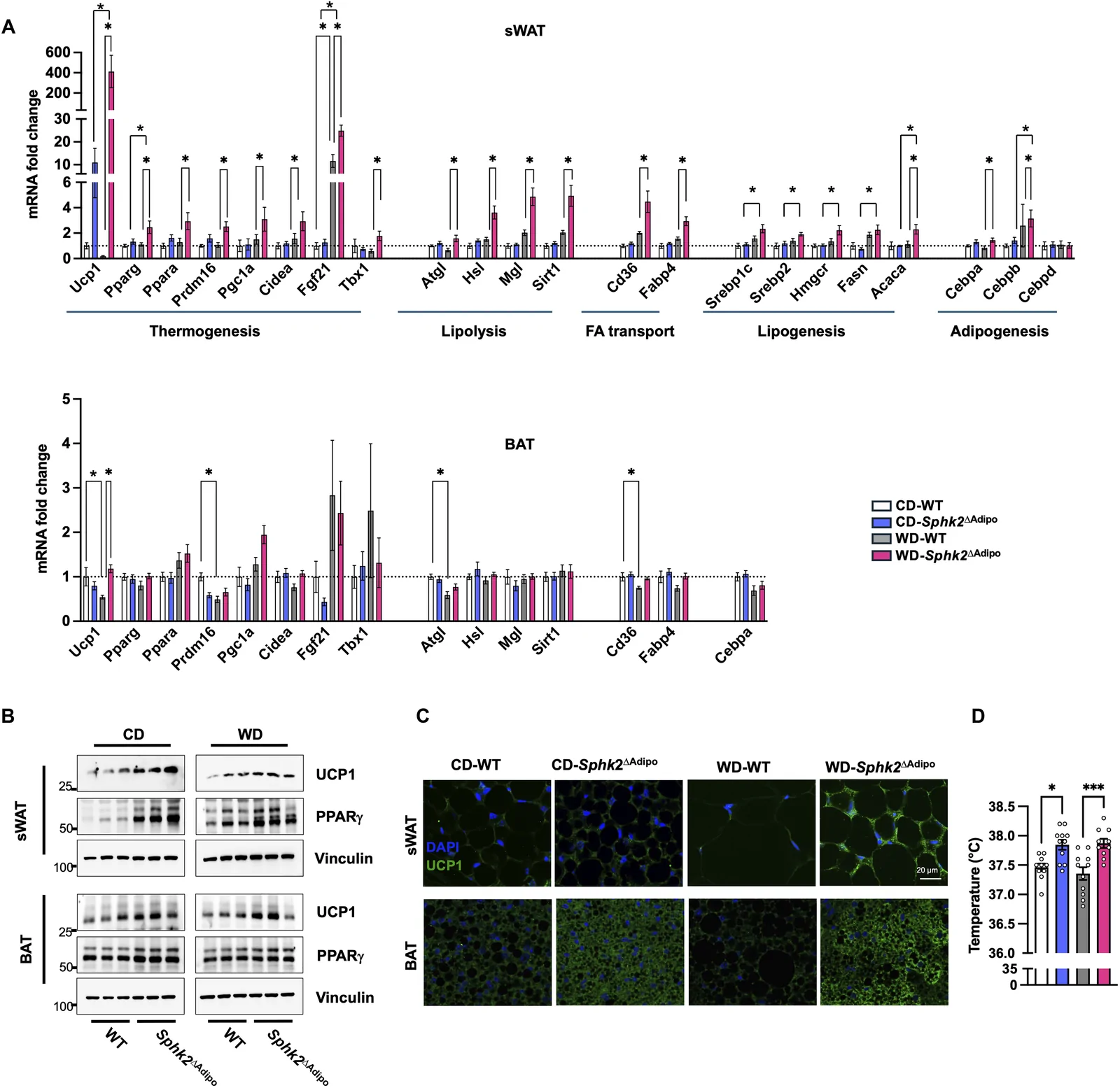
SPHK2 regulates adipocytes expression of lipolysis and thermogenesis genes in vivo. (A to D) *Sphk2*^∆Adipo^ and WT male mice were fed chow or WD for 18 weeks. (**A**) Expression of thermogenesis, lipolysis, fatty acid transport, lipogenesis, and adipogenesis genes in sWAT and in BAT, as indicated (*N* = 6). (**B**) Representative Western blots of uncoupling protein 1 (UCP1) and peroxisome proliferator–activated receptor γ (PPARγ) in sWAT and BAT. Vinculin was used as loading controls. (**C**) Representative confocal micrographs of sWAT stained for UCP1 or PPARγ and counterstained with 4′,6-diamidino-2-phenylindole (DAPI; *N* = 6). (**D**) Rectal temperatures. (*N* = 10 to 13). (A and D) Data are presented as means ± SEM; *P ≤ 0.05. One-way ANOVA test followed by Tukey’s multiple comparisons test.

Extensive analysis of key genes involved in thermogenesis, lipolysis, fatty acid transport, and lipogenesis revealed that the brown/beige fat thermogenic gene program, characterized by induction of *Ucp1*, *Pparg*, *Ppara*, *Prdm16*, *Pgc1a*, *Cidea*, and *Fgf21* and the beige cell maker *Tbx1*, was markedly stimulated in the sWAT, but only marginally in the BAT of WD-fed *Sphk2*^∆Adipo^ mice (Fig. 5A). For example, *Ucp1* was increased more than 400-fold in sWAT and only twofold in BAT. Likewise, *Fgf21*, known to promote thermogenic gene expression as an autocrine factor in adipocytes (*53*), was up-regulated by more than 20-fold in sWAT and only 2.5-fold in BAT (Fig. 5A). Similar changes, although not as marked, were observed in sWAT from WD-fed *Sphk2*^∆Adipo^ female mice (fig. S5A). Transcripts of the general markers of lipolysis and fatty acid transport were also increased in sWAT but not in BAT, whereas lipogenesis genes were largely unaltered (Fig. 5A and fig. S5A). Intriguingly, deletion of *Sphk2* also increased *Ucp1* expression even in nonobese male and female mice on a chow diet (Fig. 5A and fig. S5A). We verified enhanced expression of UCP1 and peroxisome proliferator–activated receptor γ (PPARγ), a nuclear transcriptional regulator of thermogenesis, lipid metabolism, and insulin sensitivity (*54*), by immunoblots (Fig. 5B and fig. S5B) and confocal immunofluorescence microscopy (Fig. 5C and fig. S5, C and D). Large changes were again confined to sWAT, although expectedly higher basal levels of UCP1 were found in the BAT (Fig. 5, B and C, and fig. S5, B and C). Mice maintain a normal body temperature on WD. Nevertheless, as anticipated, the increased UCP1 influenced heat production as adipocyte-specific *Sphk2* depletion increased body temperature (Fig. 5D and fig. S5E).

### Cell-autonomous SPHK2 deletion promotes thermogenic reprogramming and mitochondrial uncoupling in primary white adipocytes

**Fig. 6.**
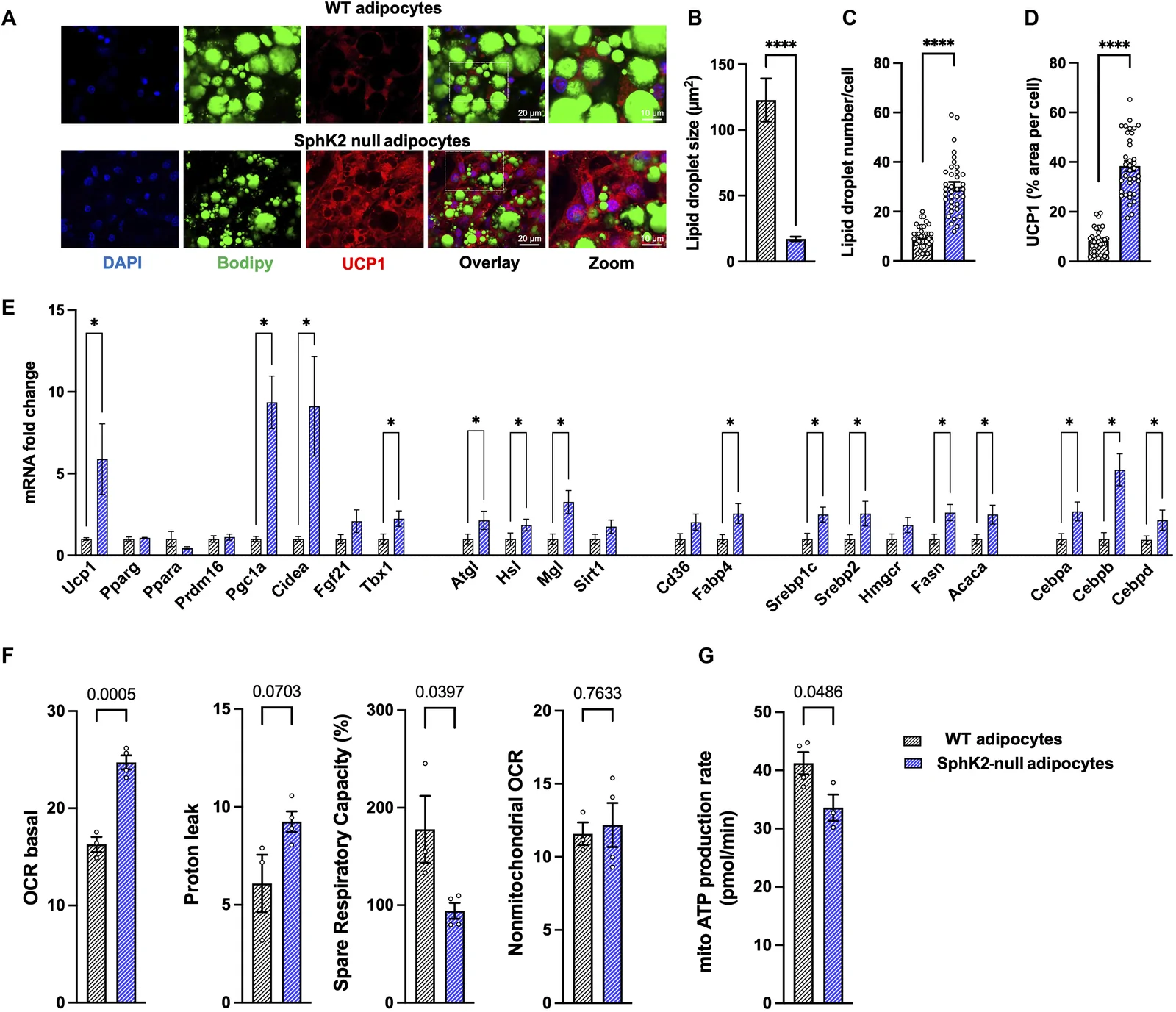
SPHK2 deficiency promotes cell-autonomous thermogenic reprogramming and enhances uncoupled mitochondrial respiration in primary adipocytes. (A to D) WT and *Sphk2*-deleted primary white adipocytes were stained for UCP1 and neutral lipids using Bodipy and counterstained with DAPI. (**A**) Representative confocal micrographs. (**B**) Lipid droplet sizes. (**C**) Numbers of lipid droplets per cell. (**D**) % UCP1-stained area per cell (*N* = 6, *n* = 6). (**E**) Expression of thermogenesis, lipolysis, fatty acid transport, lipogenesis, and adipogenesis genes in WT and *Sphk2* null primary white adipocytes (*N* = 6). (**F**) Seahorse XF Cell Mito Stress Test. Oxygen consumption rate (OCR) was measured in WT and *Sphk2*-deleted primary white adipocytes under basal conditions and following sequential injections of oligomycin (ATP synthase inhibitor), carbonyl cyanide *p*-trifluoromethoxyphenylhydrazone (uncoupler of the mitochondrial inner membrane potential), and rotenone/antimycin A (complex I and III inhibitors). Basal OCR, oligomycin-insensitive (proton leak) respiration, coupling efficiency, and nonmitochondrial OCR were calculated. (**G**) Seahorse XF Real-Time ATP Rate Assay. OCR was measured before and after oligomycin and rotenone/antimycin A injections, and mitochondrial ATP production rates were quantified. Data are presented as means ± SEM; *P ≤ 0.05 compared with WT primary adipocytes, unpaired two-tailed *t* test.

As the adipose depot is heterogeneous to examine whether the thermogenic changes in *Sphk2*^∆Adipo^ mice were confined to adipocyte-intrinsic functions without confounding effects from cross-talk with neighboring cell types, we used adipose-derived stem cells isolated from the stromal vascular fraction that were terminally differentiated to primary adipocytes in vitro. Similar to the in vivo results (Fig. 2, D and E), primary adipocytes derived from sWAT of *Sphk2*^∆Adipo^ mice had an increased number of smaller lipid droplets (Fig. 6, A to C) and enhanced expression of *Ucp1* along with increased *Pgc1*α and *Cidea*, indicating up-regulation of the thermogenic program in white adipocytes (Fig. 6, D and E). Although not as marked as in vivo, genes involved in lipolysis, fatty acid transport, lipogenesis, and adipogenesis were also up-regulated in these *Sphk2*-deleted primary adipocytes compared with WT adipocytes (Fig. 6E). These data indicate a greater potential for browning or development of beige adipocytes in cells devoid of *Sphk2*.

Seahorse XF analyses revealed that SPHK2-null primary adipocytes with increased UCP1 expression (Fig. 6, A and E) displayed significant elevation in basal oxygen consumption rate (OCR) and a trend of increased oligomycin-insensitive, ATP synthase–independent respiration (proton leak) (Fig. 6F), indicating enhanced uncoupled mitochondrial activity. Coupling efficiency was reduced while nonmitochondrial OCR remained unchanged, suggesting that a greater proportion of oxygen consumption was diverted away from ATP synthesis (Fig. 6F). Consistent with this, real-time ATP rate analysis did not show a proportional increase in ATP production rates and instead revealed a significant reduction in mitochondrial ATP production rate (Fig. 6G), confirming that the elevated respiratory flux is not used for ATP generation. Together, these findings indicate that increased UCP1 expression in SPHK2-deficient primary adipocytes results in functional mitochondrial uncoupling and supports enhanced thermogenic capacity.

### *Sphk2*^∆Adipo^ mice are highly responsive to β-adrenergic stimulation

**Fig. 7.**
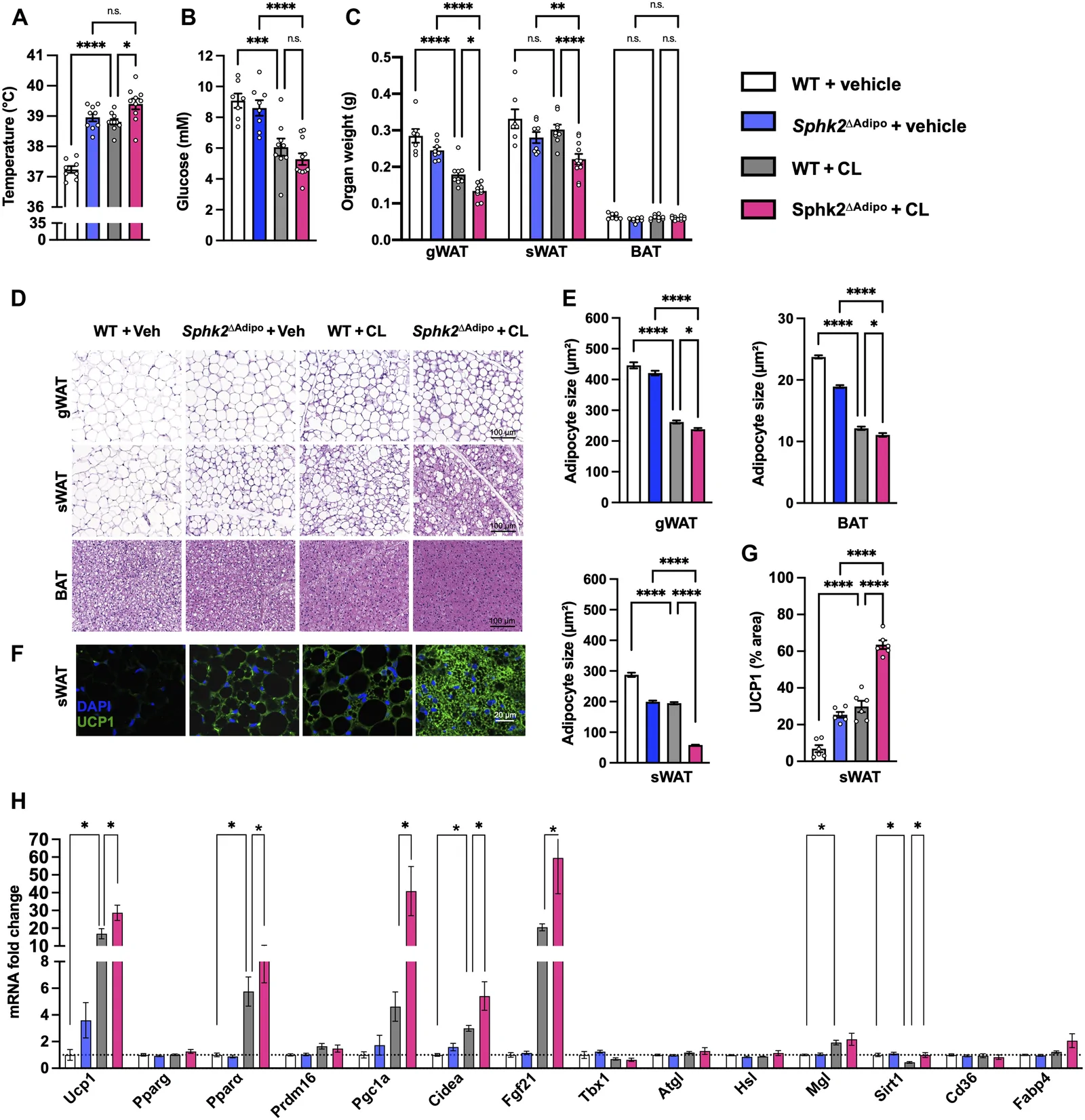
CL316243-mediated adipose browning is exacerbated by adipocyte-specific deletion of *sphk2*. (A to H) *Sphk2*^∆Adipo^ and WT male mice littermates fed chow diet were injected with vehicle or CL316243 for 7 consecutive days. (**A**) Rectal temperatures (*N* = 10 to 13). (**B**) Fasted blood glucose. (**C**) Organ weights (*N* = 7 to 11). (**D**) Representative images of hematoxylin and eosin–stained gWAT, sWAT, and BAT. (**E**) Adipocyte cell size (*N* = 6). (**F**) Representative confocal micrographs of sWAT stained for UCP1 and counterstained with DAPI. (**G**) % UCP1-stained area per field (*N* = 6). (**H**) mRNA expression of thermogenesis, lipolysis, and fatty acid transport genes in sWAT (*N* = 6). Data are presented as means ± SEM. (A to C, E, G, and H). One-way ANOVA test followed by Tukey’s multiple comparisons test. Veh, vehicle; n.s., not significant.

Browning and induction of UCP1 expression in mouse sWAT can be stimulated by pharmacological β3-adrenoreceptor agonists such as CL316243 that trigger lipolysis and thermogenesis (*51*). Thus, we investigated whether *Sphk2*^∆Adipo^ mice exhibit a different response to β-adrenergic receptor stimulation compared with WT mice. Consistent with previous reports (*55*, *56*), CL316243 treatment led to increased body temperature, reduction in blood glucose, as well as reductions in gWAT, and, to a lesser extent, sWAT tissue mass without affecting BAT weight (Fig. 7, A to C, and fig. S6, A to C). Although adipocyte-specific *Sphk2* deletion did not change the effect of CL316243 on blood glucose, it further increased body temperature and reduced gWAT and sWAT masses, indicative of exacerbated browning (Fig. 7, A to C, and fig. S6, A to C).

Microscopic evaluation showed that CL316243 reduced size of WT adipocytes, regardless of the depot or sex (Fig. 7, D and E, and fig. S6, D and E). Adipocytes of CL316243-treated *Sphk2*^∆Adipo^ mice were significantly smaller in all three depots, with notable size reductions in sWAT (Fig. 7, D to F, and fig. S6, D and E). Notably, in both sexes, *Sphk2*^∆Adipo^ mice had an enhanced response to CL316243 compared with their WT littermates, resulting in larger areas of UCP1–highly expressing multilocular beige adipocytes in sWAT (Fig. 7, F and G, and fig. S6, F and G). Consistent with sWAT adipose browning, CL316243 treatment of WT mice resulted in a significant increase in key thermogenic genes, and *Sphk2* deletion further augmented the expression of *Ucp1*, *Pgc1*α, *Cidea*, *Fgf21*, and *Sirt1* (Fig. 7H).

### WD enhances nuclear localization of SPHK2 in subcutaneous white adipose

**Fig. 8.**
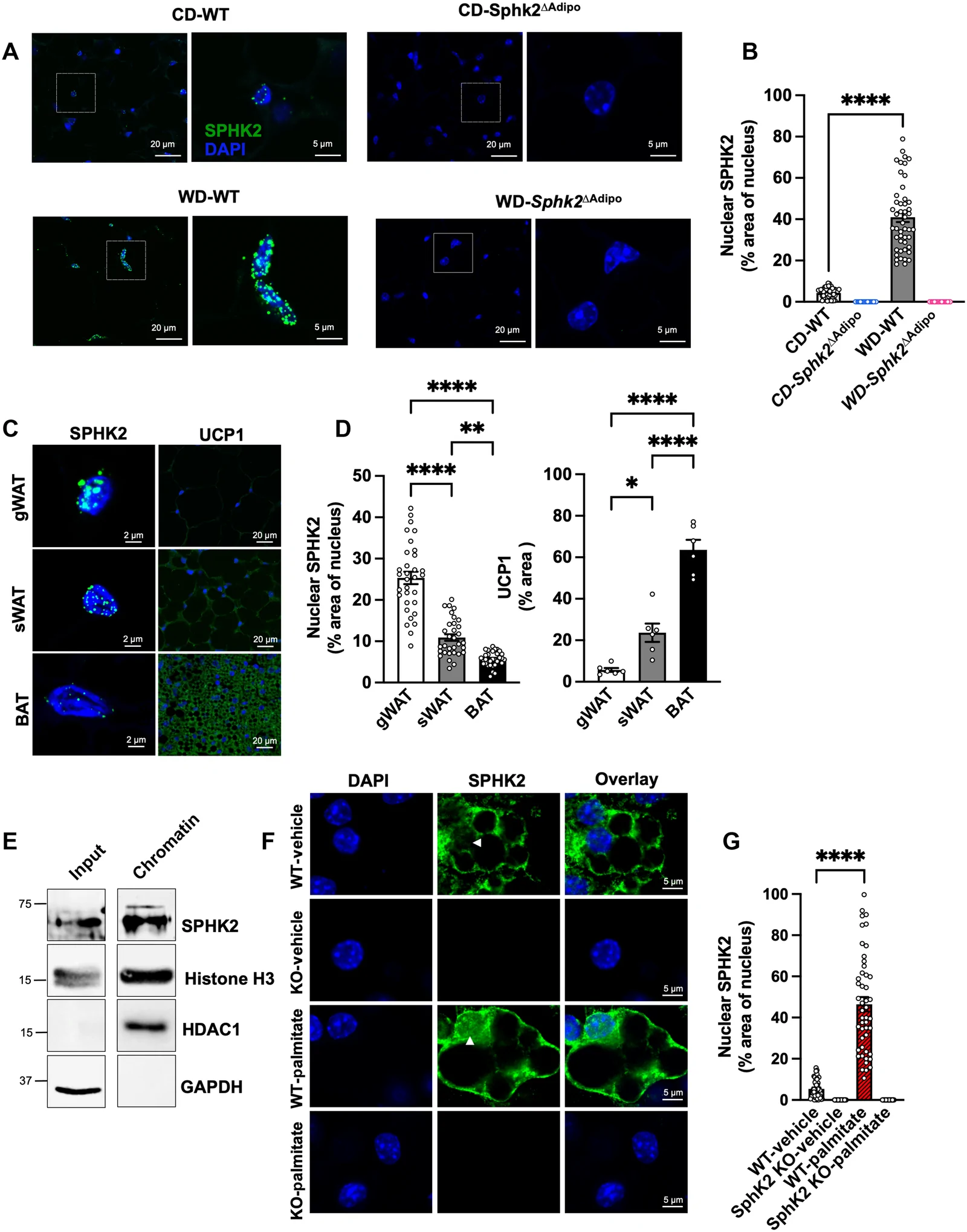
WD enhances nuclear localization of SPHK2 in SWAT. (**A**) Representative confocal micrographs of sWAT from 18 week chow or WD fed *Sphk2*^∆Adipo^ and WT male mice were stained for SPHK2 and counterstained with DAPI and then quantified (**B**) as % SPHK2-stained area per nuclei (*N* = 6, *n* = 8). Data are presented as means ± SEM; ****P ≤ 0.0001 compared with WT littermates fed chow diet, unpaired two-tailed *t* test. (**C**) Representative confocal micrographs of sWAT, gWAT, and BAT from WT mice stained for SPHK2 or UCP1 and counterstained with DAPI and were quantified (**D**) as % SPHK2-stained area per nuclei or as % UCP1-stained area per field (*N* = 4, *n* = 8). (**E**) Chromatin fractions from WT primary white adipocytes were separated by SDS–polyacrylamide gel electrophoresis (PAGE) and immunoblotted with SPHK2-specific antibody. Antibodies against histone H3 and glyceraldehyde-3-phosphate dehydrogenase (GAPDH) were used as markers for chromatin and cytosol, respectively. One percent input from these cell lysates was included as a positive control. (**F**) Representative immunofluorescence images of WT or *Sphk2* knockout (KO) primary adipocytes treated with vehicle or palmitate (500 μM) for 24 hours and were quantified (**G**) as % SPHK2-stained area per nuclei (*N* = 6, *n* = 8). Scale bars, 5 μm. (D, E, and G) Data are presented as means ± SEM; one-way ANOVA test followed by Tukey’s multiple comparisons test.

To understand how *Sphk2* deletion influenced adipose tissue function and markedly enhanced thermogenic capacity (Figs. 4 to 7), we next examined the localization of SPHK2 in sWAT from WD-fed mice. In many cells, SPHK2 was mainly localized to the nucleus or shuttled between the cytosol and the nucleus consistent with its nuclear localization and export signals (*57*–*59*). As expected, no SPHK2 staining was observed in *Sphk2*^∆Adipo^ adipocytes (Fig. 8A). WD feeding led to a marked increase of SPHK2 localized in the nucleus of sWAT adipocytes (Fig. 8, A and B). We also compared SPHK2 localization between the different adipose tissues (Fig. 8, C and D). Nuclear SPHK2 abundance corresponded with total SPHK2 protein levels (Fig. 1) and inversely correlated with UCP1 levels (Fig. 8D), consistent with depot-specific thermogenic differences observed in vivo.

As was reported previously for other cell types (*58*, *59*), in primary white adipocytes, endogenous SPHK2 was also present in the nucleus and mainly associated with chromatin (Fig. 8, E and F). To further substantiate that lipid overload influences SPHK2 subcellular localization, primary adipocytes were treated with palmitate, a saturated fatty acid that has been shown to induce adipocyte hypertrophy and lipid accumulation and is widely used to model lipid-induced metabolic dysfunction (*60*). Palmitate treatment of primary adipocytes markedly increased nuclear SPHK2 localization (Fig. 8, F and G). These results suggest that nuclear localization of SPHK2 may be involved in the WD-suppressed thermogenic program in sWAT.

### SPHK2 promotes epigenetic repression of thermogenic genes in WAT

**Fig. 9.**
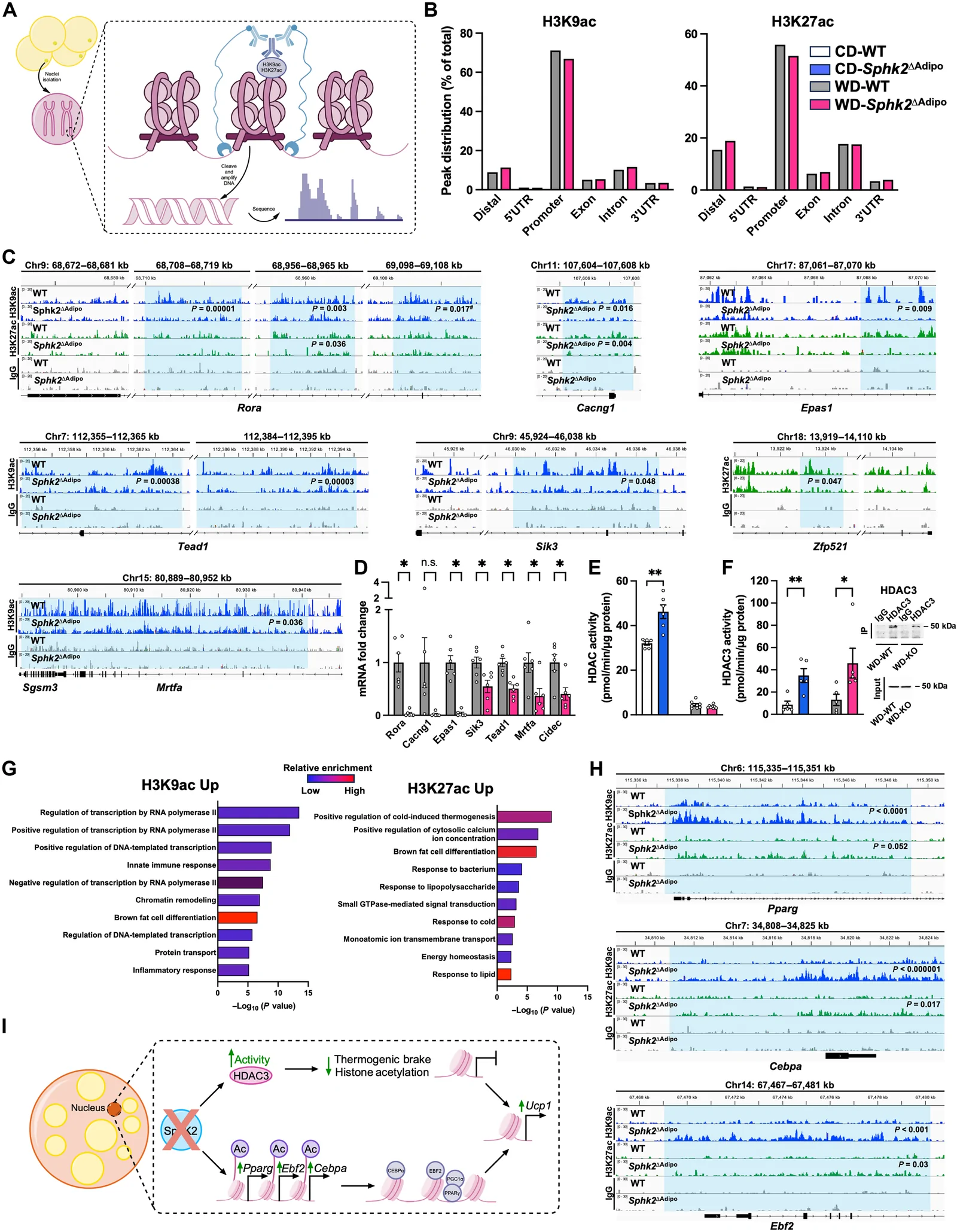
SPHK2 epigenetic regulation of subcutaneous white adipose browning. (**A**) Schematic of experimental design for CUT&Tag genome profiling of H3K9 and H3K27 acetylation from isolated sWAT nuclei. (**B**) Average proportional genome-wide H3K9ac and H3K27ac peak distribution at specific regions. 3′UTR, 3′ untranslated region; 5′UTR, 5′ untranslated region. (**C**) Representative significant reduced H3K9ac (blue tracks) and H3K27ac (green tracks) peaks for *Sphk2*^∆Adipo^ sWAT compared with WT and IgG at *Rora*, *Epas1* (*Hif-2*α), *Sik3*, *Tead1*, *Zfp521*, *Mrtfa*, and *Cacng1*, repressors of thermogenic loci. Blue-shaded boxes represent significant differentially acetylated regions (*P* ≤ 0.05 WT versus *Sphk2*^∆Adipo^ and WT versus IgG; #*P* ≤ 0.05 WT versus *Sphk2*^∆Adipo^ only; *N* = 3). (**D**) Expression of negative regulators of thermogenesis genes. (**E**) Total HDAC activity in nuclear extracts from sWAT of chow- and HFD-fed WT and *Sphk2*^ΔAdipo^ mice. HDAC activity expressed as picomoles per minute per milligram nuclear protein (pmol/min/mg). (**F**) Left: Extracts of sWAT from HFD-fed WT and *Sphk2*^ΔAdipo^ mice were immunoprecipitated with control IgG or HDAC3-specific antibody. Immunoprecipitates were washed, and HDAC3 activity was measured and expressed as pmol/min/mg HDAC3 protein. Data are presented as means ± SEM; *P ≤ 0.05. Right: Immunoprecipitated proteins were separated by SDS-PAGE and analyzed by Western blotting with HDAC3-specific antibody. (**G**) Pathway enrichment analysis for GO biological processes using genes with significantly enriched H3K9ac and H3K27ac peaks over IgG control and that have significantly increased (up) enrichment for WD-fed *Sphk2*^∆Adipo^ sWAT compared with WD-fed WT. The degree of enrichment is depicted as blue (low) to red (high) (logFC of ±0.37, *P* ≤ 0.05) (*N* = 3). (**H**) Representative significant differentially enriched H3K9ac (blue tracks) and H3K27ac (green tracks) peaks for *Sphk2*^∆Adipo^ sWAT compared with WT and IgG at *Pparg*, *Cebpa*, and *Ebf2* thermogenic loci. Blue-shaded boxes represent significant differentially acetylated regions (*P* ≤ 0.05, *N* = 3). (**I**) Summary schematic depicting the epigenetic control of white adipose browning by adipocyte SPHK2. GTPase, guanosine triphosphatase; n.s., not significant.

This prompted us to investigate the nuclear functions of SPHK2, including its known roles in modulating the recruitment of transcription factors, their cofactors, and enzymes that epigenetically regulate transcriptional programs (*58*, *59*, *61*, *62*). The elevated thermogenic program that we identified in sWAT of *Sphk2*^∆Adipo^ mice, representing browning of WAT (Fig. 4F), is known to be epigenetically regulated by changes in acetylation of histone H3 lysine 9 (H3K9ac) and lysine 27 (H3K27ac) at promoter and enhancer regions of thermogenic genes (*40*). To identify regions with altered histone acetylation and their corresponding genes potentially driving adipose browning in *Sphk2*^∆Adipo^ mice, we performed cleavage under targets and tagmentation (CUT&Tag) experiments on nuclei isolated from sWAT of WD-fed WT and *Sph*k2^∆Adipo^ mice using H3K9ac or H3K27ac antibodies (Fig. 9A). Global analysis of the proportional distribution of peaks across specific regions showed that most of peaks are located at gene promoters with no differences in the overall distribution between WT and *Sphk2*^∆Adipo^ nuclei (Fig. 9B).

We next examined promoters and potential distal enhancer regions with differentially marked H3K9ac and H3K27ac. Compared with those in WT sWAT, we identified a larger number of promoters and distal enhancer regions (>2 kb from transcription start site) in *Sphk2*-deleted sWAT with significant changes in histone acetylation at both lysine residues (promoters: H3K9ac, 1297 up and 183 down; and H3K27ac, 277 up and 29 down; enhancers: H3K9ac, 679 up and 267 down; and H3K27ac, 348 up and 74 down). Because it was previously shown that S1P formed in the nucleus by nuclear SPHK2 inhibits class I histone deacetylases (HDACs), including HDAC1, HDAC2, HDAC3, and HDAC8 (*58*, *59*, *63*), which remove acetyl groups from histones resulting in suppression of gene expression, we first focused on gene promoters and potential enhancers in SPHK2-deleted sWAT with significantly decreased H3 acetylation. We identified a subset of 12 genes encoding well-defined negative regulators of adipocyte thermogenesis that exhibited significantly reduced H3K9ac and/or H3K27ac at their promoters or enhancers in Sphk2-deficient sWAT. These include *Rora*, *Cacng1*, *Epas1* (*Hif-2*α), *Tead1*, *Sik3*, *Zfp521* (*P* < 0.05, logFC of <−0.37 WT versus Sphk2^ΔAdipo^), and *Mrtfa* (*P* < 0.05, logFC of <−0.25 WT versus *Sphk2*^ΔAdipo^), all of which are known repressors of thermogenic or beige adipocyte programs (Fig. 9C). We verified reduced expression of *Rora*, *Cacng1*, *Epas1*, *Sik3*, *Tead1*, *Mrtfa*, and *Cidec*, genes known to repress thermogenic or beige adipocyte programs in the sWAT of WD-fed Sphk2^ΔAdipo^ mice (Fig. 9D). However, increased HDAC1 and HDAC2 activity due to reduced nuclear S1P cannot account for these observations, as they have been reported to suppress brown fat gene expression (*64*). Instead, our findings align with previous studies identifying HDAC3 as a key regulator of brown adipose thermogenesis (*65*, *66*). To explore this possibility, we assessed the impact of adipocyte-specific *Sphk2* deletion on HDAC and HDAC3 activities. Total HDAC activity was increased in nuclear extracts from sWAT of chow-fed *Sphk2*^ΔAdipo^ mice compared with those of WT counterparts (Fig. 9E), consistent with the elevated *Ucp1* expression observed in nonobese mice maintained on a chow diet (Fig. 5A). In agreement with a previous report (*67*), HFD markedly reduced total HDAC activity (Fig. 9E). While *Sphk2* deletion did not significantly affect overall HDAC activity in this case (Fig. 9E), HDAC3 enzymatic activity in sWAT lysates from mice fed chow or HFD was significantly increased by the adipocyte-specific *Sphk2* deletion (Fig. 9F). Together, these data indicate that loss of SPHK2-dependent nuclear S1P signaling can relieve chromatin-based repression of multiple thermogenic brake genes, likely through enhanced HDAC3 activity, thereby favoring thermogenic activation and beige adipocyte formation.

Nevertheless, nuclear SPHK2/S1P can function beyond HDAC inhibition (*62*, *68*). It has been suggested that nuclear SPHK2/S1P can additionally function as a transcriptional coactivator by binding to transcription factors through its LXXLL motifs, enhancing their nuclear translocation and activation of target genes (*62*). In line with this possibility, we were intrigued to find that pathway enrichment analysis of genes with significant increases in promoter acetylation identified multiple biological processes related to gene transcription and browning of sWAT. Specifically, genes with increased H3K9ac were significantly enriched for pathways related to positive regulation of gene transcription and brown fat cell differentiation (Fig. 9G). Likewise, increased H3K27ac was found at genes enriched for positive regulation of cold-induced thermogenesis, brown fat cell differentiation, and responses to cold and lipid (Fig. 9H). We detected increased histone acetylation at the promoters of key thermogenic genes, including *Pparg*, *Cebpa*, and *Ebf2* (Fig. 9I), transcription factors that induce brown fat gene programs (*54*, *69*, *70*). Together, these data suggest that nuclear SPHK2/S1P regulates changes in histone acetylation associated with enhanced thermogenesis through HDAC3-dependent and potentially HDAC-independent mechanisms.

## DISCUSSION

The prevalence of obesity has tripled over the last 50 years, with the highest rates amongst Europeans and Americans (*1*). The pathology is believed to stem from dysregulation of white adipocyte function, the canonical role of which is the storage of excess energy in the form of TGs and cholesterol. Under chronic energy excess, such as in obesity, excessive lipid accumulation leads to lipotoxicity and ectopic fat deposition in the liver, contributing to hepatosteatosis and metabolic dysfunction, often co-occurring with MASLD/MASH. This highlights the complex interorgan signaling that becomes dysregulated under metabolic stress, which remains poorly understood. The metabolic reprogramming of energy-storing white adipocytes to thermogenic energy-expending beige/brown adipocytes, termed adipose browning, greatly influences systemic metabolism (*2*, *35*). Transcriptomic changes lead to the phenotypic switch (*40*), resulting in increased lipolysis, fatty acid β-oxidation, and thermogenesis that is predominantly driven by UCP1-dependent uncoupling of respiration to dissipate energy from substrate oxidation as heat (*43*, *44*). A deeper understanding of the molecular mechanisms regulating the thermogenic function of brown and beige adipocytes in vivo could lead to previously unidentified strategies for stimulating thermogenesis. This could offer a therapeutic opportunity for treating obesity and related comorbidities, such as type 2 diabetes and MASLD/MASH (*3*, *40*).

In this study, we found that deletion of *Sphk2* in adipocytes protected mice from diet-induced obesity and increased systemic energy expenditure. These adipocytes had reduced hypertrophy and an up-regulation of thermogenic genes, particularly *Ucp1*. This effect was attributed to the epigenetic transcriptional reprogramming of subcutaneous white adipocytes into thermogenic beige/brown adipocytes. We also observed substantial systemic changes in circulating adipokines. For example, we found that adiponectin known to improve insulin sensitivity and to reduce fatty liver was increased. Similarly, there were increases in adipose-derived Fgf21, which improves insulin sensitivity and helps to maintain overall metabolic homeostasis. As a result, *Sphk2*^∆Adipo^ mice had improved glucose tolerance and insulin sensitivity and were protected from hepatosteatosis. Together, these results suggest that adipocyte SPHK2 may be involved in interorgan signaling and systemic energy homeostasis. Nevertheless, our results do not definitively establish whether the attenuation of hepatic lipid accumulation observed in *Sphk2*^∆Adipo^ mice reflects a direct adipose-liver signaling axis mediated by SPHK2/S1P pathways or is largely secondary to the overall reduction in body weight, adiposity, and systemic metabolic stress.

In addition, deletion of *Sphk2* solely in adipocytes also reduced hypercholesterolemia in *Sphk2*^∆Adipo^ mice without affecting food intake. This is unexpected as cholesterol is primarily synthesized by the liver. It is thus conceivable that decreased circulating cholesterol in WD-fed *Sphk2*^∆Adipo^ mice may be a result of changes to interorgan signaling and hepatic reduction of de novo cholesterol biosynthesis.

The reduction in the RER and the simultaneous increase in the expression of lipolytic enzymes in sWAT of *Sphk2*^∆Adipo^ mice suggest that excess lipids from an obesogenic diet are not excessively stored by adipocytes. Instead, these lipids are likely used as a primary fuel source due to the up-regulation of thermogenic genes. This metabolic shift prevents lipid redistribution to the liver, thereby offering protection against the development of MASLD. Furthermore, our data implies that the systemic metabolic benefits reported in global *Sphk2^−/−^* mice (*20*, *21*) are due, at least, in part, to deletion of *Sphk2* in adipocytes.

Abundant evidence demonstrated that the sphingolipid metabolite ceramide accumulates in obesity and contributes to metabolic dysfunction (*4*–*6*, *8*–*11*). In adipose tissue, excessive ceramides impair insulin signaling, leading to reduced glucose uptake and increased lipolysis, which exacerbates systemic metabolic disturbances (*5*, *12*, *13*, *36*). However, most of the studies did not investigate the potential contribution of S1P, a metabolite of ceramide. Moreover, elegant studies with adipocyte-specific deletion of *Sptlc2* or *Degs1* indicate that ceramides are necessary and sufficient for diet-induced impairment of thermogenic adipocyte function (*5*, *13*). Nevertheless, these key enzymes are involved in the de novo biosynthesis of all sphingolipids and may also affect the levels of S1P, which could have contributed to the observed metabolic changes.

In this study, we showed that adipocyte-specific deletion of *Sphk2* substantially reduced levels of S1P and DHS1P in adipose and concomitantly up-regulated the thermogenic program and induced browning of sWAT. This occurred despite elevated ceramide levels likely caused by a backlog in the sphingolipid biosynthetic pathway. These findings suggest that the attenuation of diet-induced S1P in *Sphk2*^∆Adipo^ mice has a dominant effect in regulating white adipocyte function and obesity development, despite elevated ceramide levels. Moreover, the modest increase in ceramide observed in *Sphk2*-deficient adipose tissues may be insufficient to affect their functions.

Like others (*4*–*6*, *19*, *33*), we also showed that WD increased levels of S1P in adipose and circulation. In contrast to *Sphk2*-null mice that have increased blood levels of S1P (*38*), *Sphk2*^∆Adipo^ mice had notably reduced levels of S1P and DHS1P not only in adipose but also in blood and plasma. Although it was previously reported that erythrocytes and vascular endothelium are the major sources of blood S1P (*71*), our findings in plasma indicate that adipocytes significantly contribute to regulation of circulating S1P. These findings suggest that S1P and DHS1P are not produced by adipocyte SPHK1 (*19*), but predominantly by SPHK2, which is essential for maintaining homeostatic plasma S1P levels. Deletion of *Sphk2* in adipocytes also markedly suppressed WD-induced pathological increases of S1P and DHS1P. Therefore, adipocyte SPHK2 is, at least, partially responsible for the pathological increase in circulating S1P associated with obesity. Additionally, the elevated S1P levels in blood could potentially result from the effect on obesity that can be associated with elevated red blood cell counts. Notably, sustained weight loss in humans leads to increased adiponectin and reduced FGF21 and leptin, as well as more marked decrements in ceramide and S1P (*72*). Together with previous studies, this finding indicates that circulating ceramide and S1P undergo substantial alterations in response to diet-induced weight gain or loss as well as to weight-loss maintenance treatments. Moreover, there were several associations between the circulating metabokines and these bioactive sphingolipid metabolites (*72*). While these associations do not imply causation, they highlight the potential importance of shifts in the circulating metabokine-sphingolipid metabolite profiles for sustaining healthy weight loss. Our findings also suggest that the SPHK2/S1P axis may be a promising therapeutic target for treating metabolic disorders (*26*).

Adipocyte-specific deletion of *Sphk2* resulted in the differential expression of genes and smaller lipid droplets in all three adipose depots examined. However, the thermogenic response was more apparent in WAT compared with BAT and highest in sWAT that has been shown to be more susceptible to browning than the metabolically unfavorable visceral fat (*73*). The limited thermogenic response of BAT to deletion of *Sphk2* may be due to the high basal level of UCP1 and, thus, limited capacity to increase further. Similarly, in several other genetically modified mice models, WAT browning was induced with only minimal effects on BAT, demonstrating that specific transcriptional and signaling pathways regulate white adipocyte browning independently of classical BAT function (*74*). We also observed a significant down-regulation in inflammatory response pathways exclusively in BAT that is known to correlate with thermogenic activity (*75*). Previous studies analogously demonstrated the inflammatory-suppressive role of *Sphk2* deletion (*76*). Therefore, we cannot rule out that reduced inflammation in BAT may also contribute to the obesity-resistant phenotype of *Sphk2*^∆Adipo^ mice. Because *Sphk2*^∆Adipo^ mice exhibited reduced sWAT adipocyte size and elevated *Ucp1* expression even on a chow diet, though the effect was far less pronounced than in WD-fed mice (10-fold versus 400-fold), it is possible that SPHK2 could also play a physiological role in adipocyte metabolic functions.

While adipocyte-specific deletion of *Sphk2* provided protection from diet-induced obesity and insulin resistance, deletion of *Sphk1* in adipocytes has been reported to cause hypertrophy, impaired lipolysis, and exacerbate diet-induced obesity and MASLD by increasing inflammation in WAT (*19*). Moreover, SPHK1 by maintaining lysosomal integrity in brown adipocytes was reported to be involved in TG breakdown (*18*). Thus, SPHK1 and SPHK2 appear to have isoform-specific roles in adipocytes, most likely due to their different subcellular localization and production of S1P (*71*). SPHK1 is mainly a cytosolic enzyme and is translocated to the plasma membrane in response to stimuli, whereas SPHK2 has been shown to have important functions in the nucleus (*58*). We observed that WD markedly increased nuclear localization of SPHK2. In the nucleus, SPHK2 can interact with class I HDACs, transcription factors, or coactivator complexes to regulate gene expression, in part by modulating histone acetylation at actively transcribed genes (*58*, *59*, *61*, *62*). Class I HDACs, despite their structural similarities, have opposing effects on *Ucp1* expression (*64*–*66*). HDAC1 and HDAC2 primarily act as repressors of thermogenic gene programs (*64*), whereas HDAC3 is essential for maintaining thermogenic competence in the BAT (*65*, *66*). Consistent with its role as a corepressor, deletion of HDAC3 leads to the induction of numerous genes, including canonical targets of nuclear receptor-mediated repression. However, the loss of HDAC3 also results in marked down-regulation of key thermogenic and metabolic genes, including *Ucp1* and *Pgc1*α, as well as mitochondrial oxidative phosphorylation genes (*65*). This dysregulation impairs mitochondrial respiration and mice develop severe hypothermia. Mechanistically, it was suggested that HDAC3 functions as a critical coactivator of ERR-driven thermogenic gene transcription in BAT and the BAT-specific EBF2. Beyond its posttranslational regulation of PGC-1α, HDAC3 has been shown to occupy the *Pgc-1*α locus together with ERRα at highly active enhancer regions of *Ucp1*. These enhancer activities are diminished in the absence of HDAC3, suggesting that HDAC3 can function as a transcriptional coactivator at specific loci, including *Pgc-1*α and *Ucp1* (*65*).

Intriguingly adipocyte-specific *Sphk2* deletion increased HDAC3 enzymatic activity in sWAT after HFD feeding. We identified a subset of several genes encoding well-defined negative regulators of adipocyte thermogenesis that exhibited significantly reduced H3K9ac and/or H3K27ac in the absence of SPHK2. These included *Rora*, a transcription factor that represses *Ucp1* and attenuates adaptive thermogenesis (*77*); *Epas1* (*Hif-2*α), an endogenous inhibitor of beige adipocyte activation that restrains cyclic adenosine 3′,5′-monophosphate (cAMP)–dependent protein kinase (PKA)–dependent thermogenic signaling (*78*); *Sik3*, a kinase acting downstream of cAMP/PKA to inhibit BAT thermogenic gene expression and browning of WAT (*79*); and *Tead1* (TEA domain transcription factor 1), part of a repressor complex, primarily functions to suppress the thermogenic program and *Ucp1* gene expression and inhibits the development of thermogenic beige adipocytes within WAT (*80*). In addition, we observed reduced histone acetylation at *Mrtfa* (myocardin-related transcription factor A), which acts as a negative regulator of adipocyte browning and beige fat formation (*81*, *82*). Last, decreased promoter acetylation was detected at *Cacng1* (calcium voltage-gated channel auxiliary subunit gamma 1) recently identified in an unbiased CRISPR screen as a lineage repressor of beige adipocytes, where its genetic loss enhances thermogenic marker expression and WAT beiging (*83*). A reduction in histone acetylation at *Zfp521* was also observed. ZFP521 acts as a chromatin-associated repressor of beige/brown adipocyte commitment by inhibiting *Ebf2* and *Prdm16*, and its depletion enhances *Ucp1* expression and beige adipocyte recruitment (*84*). These results suggest that loss of SPHK2-dependent events can promote chromatin-mediated repression of key thermogenic inhibitory genes. This effect may be mediated through enhanced HDAC3 activity, thereby promoting thermogenic activation and facilitating beige adipocyte formation.

Additionally, SPHK2 may bind to transcription factors such as the aryl hydrocarbon receptor through its LXXLL motifs, influencing their nuclear translocation and activation of target genes (*62*). A critical event in transcriptional control of white adipose browning is epigenetic acquisition of histone acetylation on H3K9 and H3K27 at promotor regions of thermogenic genes (*40*, *85*). In line with this, sWAT from WD-fed *Sphk2*^∆Adipo^ mice exhibited increased H3K9ac and H3K27ac at gene promoters related to brown fat cell differentiation and response to cold, including the key thermogenic genes *Pparg*, *Cebpa*, and *Ebf2*. Browning of white adipose requires the recruitment of master regulators such as PPARγ, CCAAT/enhancer binding α, and PRDM16 transcription factors and the coactivator PGC1α (*40*). In addition, EBF2 acts as a pioneering transcription factor that allows PPARγ to bind and activate thermogenic genes, including *Ucp1* (*86*). sWAT from *Sphk2*^∆Adipo^ mice exhibit significantly increased expression of *Pgc1a*, *Pparg*, and *Prdm16* as well as their downstream targets, particularly *Ucp1*, linking increased epigenetic histone acetylation to thermogenic gene expression. Thus, nuclear localization of Sphk2 and S1P may also influence histone acetylation through HDAC-independent mechanisms, such as altered recruitment of transcriptional complexes or changes in chromatin accessibility.

Our study has several limitations. To date, the feasibility of detecting SPHK2 within complexes bound to DNA of mouse chromatin has been limited. We were unable to perform CUT&Tag experiments to test direct chromatin binding of SPHK2 as antibodies suitable for endogenous chromatin immunoprecipitation–based assays are now not available for mouse SPHK2, where validated reagents have been limited to immunoprecipitation. Consequently, while our CUT&Tag analyses reveal changes in H3K9ac and H3K27ac at promoters and enhancers of thermogenic and thermogenic-repressor genes in *Sphk2*^ΔAdipo^ sWAT, these findings do not directly demonstrate that SPHK2 itself functions as the epigenetic regulator responsible for these chromatin modifications. Further studies are needed to elucidate the precise molecular mechanisms by which nuclear SPHK2/S1P signaling regulates chromatin remodeling and transcriptional programs governing adipose browning.

Our study identifies a previously unrecognized role for SPHK2 in the regulation of adipose tissue thermogenesis, browning particularly in sWAT, and whole-body metabolic homeostasis. Furthermore, adipocyte SPHK2 appears to contribute, at least, in part, to the maintenance of circulating S1P homeostasis. Given that *Sphk2*^∆Adipo^ obese mice have reduced circulating S1P levels, the observed protection against systemic metabolic dysregulation may involve adipose-mediated regulation of circulating sphingolipids, in addition to adipokine signaling, as previously proposed (*4*, *5*, *9*, *12*).

## MATERIALS AND METHODS

### Animal model

C57Bl/6J *Sphk2*^Flox/Flox^;*Adipoq*^Cre/WT^ mice were generated by backcrossing B6.FVB-Tg(*Adipoq*-cre)1Evdr/J (no. 028020, the Jackson Laboratory) with *Sphk2*^Flox/Flox^ (WT) mice harboring LoxP sites flanking *Sphk2* exons 4 to 6 (*38*) to achieve deletion of *Sphk2* under the control of the *AdiopQ* promoter exclusively expressed in adipocytes. The Sphk2^Flox/Flox^ colony was maintained by breeding *Sphk2*^Flox/Flox^ male and female pairs. WT and *Sphk2*^∆Adipo^ littermates used for experimentation were subsequently generated by breeding C57BL/6J male homozygous Sphk2^Flox/Flox^ mice with C57BL/6J *Sphk2*^Flox/Flox^;*Adipoq*^Cre/WT^ females described above to produce WT and *Sphk2*^∆Adipo^ mice of both sexes. WT and *Sphk2*^∆Adipo^ 4-week-old littermates were weaned and randomly assigned to experimental groups for diet studies or used at 5 weeks old for in vitro primary adipocyte cell culture. Mice were fed standard rodent chow (Harlan TD.7912, Envigo) with regular water or WD (42% kcal milk fat, 42.7% kcal from sucrose-enriched carbohydrates, 15.2% kcal from protein, and 0.2% cholesterol) (Harlan TD.88137) supplemented with sugar drinking water [d-fructose (23.1 g/liter; no. A17718.01, Fisher) and d-glucose (18.9 g/liter; no. D16-10, Fisher)] ad libitum for 18 weeks. Mice were housed in the animal care facility at Virginia Commonwealth University under regulated humidity (50 to 60%), temperature (21° to 23°C), and 12-hour dark/light cycles. On the day of tissue collection, mice were fasted for 6 hours (7:00 a.m. to 1:00 p.m.) and euthanized by 5% isoflurane (no. 11695067772, Covetrus) inhalation, and blood was collected via cardiac puncture with heparinized syringes before being perfused transcardially with phosphate-buffered saline (PBS: no. 10010023, Fisher) containing 0.1% heparin (no. 375095, Sigma-Aldrich). All animal procedures and protocols had been approved by the Virginia Commonwealth University Institutional Animal Care and Use Committee protocol number AM10148, which is accredited by the Association for Assessment and Accreditation of Laboratory Animal Care.

### Reagents and compounds

All reagents and compounds were from general commercial sources unless otherwise stated in the following: CL316243 (no. 1499, Tocris), collagenase (no. C6885, Sigma-Aldrich), Dulbecco’s modified Eagle’s medium (DMEM)/F12 (1:1) (no. 10-090-CV, Corning), fetal bovine serum [no. S11150H (lot J22204), R&D Systems], gelatin (2%) (no. G1393, Sigma-Aldrich), mouse insulin (sc-360248, Santa Cruz Biotechnology), isobutylmethylxanthine (no. BMLPD1401000, Enzo), rosiglitazone (no. 71740, Cayman Chemical), triiodothyronine (T3) (no. T6397, Sigma-Aldrich), indomethacin (no. 17378, Sigma-Aldrich), heat induced epitope retrieval (HIER) antigen-retrieval buffer (no. ab208572, Abcam), VECTASHIELD Vibrance antifade mounting medium containing 4′,6-diamidino-2-phenylindole (DAPI; no. H-1800, Vector Laboratories), BODIPY 493/503 (no. D3922, Sigma-Aldrich), hematoxylin (no. H-3502, Vector Laboratories), High-Capacity cDNA Reverse Transcription Kit (no. 4368814, Fisher), SYBR (no. 4367659, Fisher), deoxyribonuclease I (DNase I; no. E1011, Zymo Research), Minute Single Nuclei and Cytosol Isolation Kit (no. AN-029, Invent Biotechnologies), Hyperactive Universal CUT&Tag Assay Kit for Illumina Pro (no. TD904, Vazyme), dual-index adapters (no. TD202, Vazyme), VAHTS DNA Clean Beads (no. N411, Vazyme), dsDNA kit (no. 5067-4626, Agilent), dUTP-based stranded NEBNext Ultra II Directional RNA Library Prep Kit for Illumina (no. E7760S, New England Biolabs), chow diet (Harlan TD.7912, Envigo), and WD (Harlan TD.88137, Envigo).

### Antibodies

Working dilutions of antibodies were 1:1000 for Western blot (peroxidase-conjugated secondaries, 1:5000), 1:200 for immunohistochemistry, 1:100 for immunocytochemistry, and 1:50 for CUT&Tag. The following antibody were used: anti-UCP1 (no. ab10983, Abcam, RRID:AB_2241462), anti-PPARγ (no. 2435, Cell Signaling Technology, RRID:AB_2166051), anti-SPHK2 (no. SP4621, PhosphoSolutions, RRID:AB_2619719), anti-Vinculin (no. 13901, Cell Signaling Technology, RRID:AB_2728768), anti-SPHK1 (no. ab46719, Abcam, RRID:AB_778045), anti-SPHK2 (no. 17069-a-AP, Proteintech, RRID:AB_10598479), anti-H3K27ac (no. ab4729, Abcam, RRID:AB_2118291), anti-H3K9ac (no. ab10812, Abcam, RRID:AB_297491), immunoglobulin G (IgG) isotype control (no. ab171870, Abcam, RRID:AB_2687657), anti-rabbit IgG (H+L) (no. ab6072, Abcam, RRID:AB_956012), anti-rabbit Alexa Flour 488 (no. A11008, Fisher, RRID:AB_143165), anti-rabbit Alexa Flour 555 (no. A21428, Fisher, RRID:AB_2535849), peroxidase-conjugated goat anti-rabbit secondary antibody (no. 111-035-144, Jackson ImmunoResearch, RRID:AB_2307391), and peroxidase-conjugated goat anti-mouse secondary antibody (no. 115-035-166, Jackson ImmunoResearch, RRID:AB_2338511).

### Mouse genotyping

Genomic DNA was isolated from ear clippings or tissue as previously described (*87*), and 3 μl was used for PCR with GoTaq Master Mix (no. M712C, Promega) for identification of *Sphk2*^Flox/Flox^ or *Adipoq*^Cre/WT^ expression. The following primers were used: *Adipoq*^*Cre*/WT^: forward ACGGACAGAAGCATTTTCCA and reverse GGATGTGCCATGTGAGTCTG; and *Sphk2*^Flox/Flox^: forward GAGGCTGAGAATGCCAGGTACAAC, reverse 1 TCTCACTGGTGCTAAGACAAAGCG, and reverse 2 GCTCACTGTGAATGTCCACATCTG. PCR products were separated on a 2% agarose gel to identify the *Adipoq*^Cre/WT^ transgene by a band of 200 base pairs and either *Sphk2*^Flox/Flox^ by a band of 391 base pairs or *Sphk2* deletion (Sphk2^Flox/Flox^;*Adipoq*^Cre/WT^) by a band of 560 base pairs.

### CL316243 treatment

Ten-week-old WT and *Sphk2*^∆Adipo^ chow-fed littermates were injected with vehicle (no. 64938-009-01, SALJET) or CL316243 (no. 1499, Tocris) intraperitoneally at a dose of 1 mg/kg per day at 9:00 a.m. daily for 7 consecutive days. On the seventh day, mice were fasted for 6 hours (7:00 a.m. to 1:00 p.m.) while continuing to receive treatment at 9:00 a.m., and fasted glucose and rectal temperature were measured before euthanasia by 5% isoflurane inhalation.

### Body composition analysis

After 18 weeks of chow or WD feeding, mice were weighed and restrained, and body composition was analyzed by LF90II time-domain NMR minispec (Bruker Optik, Massachusetts) to obtain fat, fluid, and lean masses.

### Whole-body metabolic phenotyping

After 16 weeks of WD feeding, mice were individually housed and acclimatized in metabolic chambers (PhenoMaster, TSE Systems) for 48 hours under regulated humidity (50%), temperature (23°C), and 12-hour dark/light cycles (70% light intensity). Measurements of water consumption, food intake, energy expenditure, VO_2_, VCO_2_, and RER for dark and light cycles were measured during 4 subsequent days. Exported raw data were analyzed using CalR (https://CalRapp.org/) (*88*).

### Body temperature

After 18 weeks of WD feeding or 7 days of CL316243 treatment, mice were fasted for 6 hours and restrained, and body temperature was measured with a lubricated rectal probe (no. 20250-92, Cole-Parmer).

### Tissue, blood, and plasma analyses

After chow or WD feeding for 18 weeks, mice were fasted for 6 hours (7:00 a.m. to 1:00 p.m.) and euthanized by 5% isoflurane inhalation, and blood was drawn via cardiac puncture with heparin-coated syringes. The plasma fraction was collected for downstream analysis by centrifuging whole blood at 2000*g* for 15 min at 4°C. Liver, gonadal, subcutaneous, and brown adipose were excised, weighed, and a portion snap-frozen in liquid N_2_. A portion of tissue was processed for embedding as detailed in the respective methods section. Fed and fasted blood glucose was measured from a nick in the tail vein using Nipro TRUEtrack Blood Glucose Meter (no. B0727V3XQX, Amazon). Fasted circulating concentrations of plasma free fatty acids, total cholesterol, insulin, leptin, and adiponectin were quantified by the Baylor College of Medicine, Mouse Metabolism and Phenotyping Core. Fasted circulating plasma glucagon was measured by enzyme-linked immunosorbent assay using manufacturer’s instructions (no. 81518, Crystal Chem).

### Glucose and insulin tolerance testing

After chow or WD feeding for 15 weeks, mice were fasted either for 4 hours (7:00 a.m. to 11:00 a.m.) for insulin tolerance testing or for 6 hours (7:00 a.m. to 1:00 p.m.) for glucose tolerance testing. For both assays, baseline blood glucose was measured from the tail vein using the Nipro TRUEtrack Blood Glucose Meter (no. B0727V3XQX, Amazon). Insulin (0.75 U/kg; no. I9278, Sigma-Aldrich) or glucose (1.5 mg/g; no. D16-10, Fisher) in PBS (no. 10010023, Fisher) was filter sterilized (no. SLGP033RS, Sigma-Aldrich) and injected intraperitoneally for insulin and glucose tolerance testing, respectively. Blood glucose was measured at 15, 30, 60, 90, and 120 min postinjection for both assays. HOMA-IR was calculated using the following equation: HOMA-IR = [fasted insulin (nanograms per milliliter) * fasted glucose (millimolar)]/22.5.

### Histology and immunohistochemistry

Excised adipose and liver sections were fixed in 10% neutral buffered formalin (no. 245684, Fisher) for 24 hours with agitation at 4°C, embedded in paraffin, and stained with hematoxylin and eosin by the Virginia Commonwealth University Tissue and Data Acquisition and Analysis Core. Alternatively, tissues were subsequently submerged in 30% sucrose (no. S5-500, Fisher) for 48 hours with agitation at 4°C and frozen in Tissue-Tek optimal cutting temperature solution containing sucrose (2:1; 20% sucrose λ) (no. 4583, Sakura). For oil red O staining, 10-μm liver cryosections were left to thaw for 30 min before submerging in 60% isopropanol (no. 383920010, Thermo Fisher Scientific). Slides were then incubated with 0.3% oil red O solution (no. O0625, Sigma-Aldrich) for 15 min at room temperature before sequential submersions in 60% isopropanol and distilled H_2_O. Sections were counterstained with hematoxylin for 2 min and submerged in H_2_O before bluing reagent (no. H-3502, Vector Laboratories) was added for 15 s. Sections were then thoroughly washed by submerging 10 times in H_2_O and mounted with CC/mount (no. C9368, Sigma-Aldrich).

For immunohistochemistry, adipose paraffin sections were dewaxed and rehydrated by sequential 5-min-long submersions at room temperature: twice in 100% Histo-Clear (no. 5089990147, Fisher) and Histo-Clear/ethanol (90:10), twice in 100% ethanol, twice in 95% ethanol, twice in 80% ethanol, and twice in 70% ethanol. Sections were then washed twice in H_2_O and then submerged in 98°C HIER antigen-retrieval buffer (no. ab208572, Abcam), both for 20 min each. Slides were then washed three times in PBS, fixed in ice-cold methanol at −20°C for 15 min, and washed a further three times in PBS for 10 min each. Sections were blocked and permeabilized in blocking/permeabilization buffer, consisting of PBS containing 5% normal goat serum (no. 31872, Fisher), 1% bovine serum albumin (BSA; no. A7906, Sigma-Aldrich), and 0.4% Triton X-100 (no. AAA16046AE, Fisher) for 1 hour at room temperature in a humidified chamber. Sections were subsequently incubated with primary antibodies all diluted in blocking/permeabilization buffer overnight in a humidified chamber at 4°C. Sections were washed three times in PBS for 10 min each and incubated with fluorescent secondary antibodies diluted in blocking/permeabilization buffer for 1 hour in a dark/humidified chamber and mounted with VECTASHIELD Vibrance antifade mounting medium containing DAPI (no. H-1800, Vector Laboratories).

Immunohistochemical bright-field micrographs were captured using a BZ-X810 fluorescence microscope (no. BZ-X810, Keyence), and ImageJ was used to quantify percentage of oil red O staining. Adipocyte size was automatically calculated from 20× bright-field micrographs of hematoxylin and eosin–stained adipose using the Adiposoft plugin for ImageJ (*89*). Confocal immunofluorescence micrographs were captured using an inverted Zeiss LSM880 microscope described below.

### Culture and differentiation of murine primary adipocytes

Subcutaneous white inguinal adipose depots were excised from 5-week-old male mice (*N* = 1 indicates two fat pads combined from one mouse per culture), briefly washed in Hanks’ balanced salt solution (HBSS; no. 14175095, Fisher) containing 1% antibiotic-antimycotic (no. A5955, Sigma-Aldrich), finely minced, and digested in 4 ml of HBSS digestion buffer containing 1% antibiotic-antimycotic (no. A5955, Sigma-Aldrich), 5 mM CaCl_2_ (no. 349610250, Fisher), 100 mM Hepes (no. H0887, Sigma-Aldrich), collagenase (3 mg/ml; no. C6885, Sigma-Aldrich), and BSA (15 mg/ml; no. A7906, Sigma-Aldrich) for 1 hour at 37°C with agitation. Once a homogenous slurry formed, the collagenase was neutralized by adding equal volumes of DMEM/F12 (1:1) (no. 10-090-CV, Corning) containing 1% antibiotic-antimycotic (no. A5955, Sigma-Aldrich), 10% fetal bovine serum [no. S11150H (lot J22204), R&D Systems], and 1% GlutaMAX (no. 35050061, Gibco) (stromal vascular cell culture medium); inverted five times; and filtered through a 100-μm cell strainer to remove debris and undigested tissue (no. CLS431752, Corning). The cell strainer was rinsed with a further 4 ml of stromal vascular cell culture medium, and the flowthrough was centrifuged at 1200*g* for 5 min. The supernatant containing mature adipocytes was carefully aspirated, and the pellet was resuspended in 5 ml of stromal vascular cell culture medium before filtering through a 40-μm cell strainer (no. CLS431750, Corning). The strainer was rinsed with a further 2 ml of medium, and the flowthrough was centrifuged at 1200*g* for 5 min. The supernatant was aspirated, and the stromal vascular fraction single-cell suspension containing adipose-derived stem cells was resuspended in 6 ml of stromal vascular cell culture medium (one mouse plated across six wells of a 24-well plate achieved confluency after 7 days) and plated on 0.2% gelatin-coated plates, replacing medium every 2 days for a total of 7 days until confluent at 37°C and 5% CO_2_.

Once adipose-derived stem cells reached confluence, medium was changed to induction medium for 48 hours depending on originating depot. For white adipocytes (from subcutaneous white inguinal adipose), induction medium contained insulin (10 μg/ml; sc-360248, Santa Cruz Biotechnology), 1 μM dexamethasone (no. D4902, Sigma-Aldrich), 0.5 μM isobutylmethylxanthine (no. BMLPD1401000, Enzo), and 1 μM rosiglitazone (no. 71740, Cayman Chemical) in stromal vascular cell culture medium. After 48 hours, induction medium was changed to maintenance medium containing insulin (10 μg/ml) and 1 μM rosiglitazone for 6 days, replacing medium every 2 days.

### Chromatin fractionation

Chromatin-bound nuclear proteins were extracted from differentiated primary adipocytes isolated from sWAT with a protein fractionation kit (no. 78840, Thermo Scientific) with minor modifications. Stromal vascular cells were plated and differentiated in 100-mm plates, then trypsinized, and centrifuged at 500*g* for 5 min at 4°C. Cells were briefly washed with ice-cold BuPH-PBS (no. 28372, Thermo Scientific), and 1 × 10^7^ cells were transferred to a microcentrifuge tube to obtain a packed cell volume of 100 μl. Cells were pelleted at 500*g* for 3 min at 4°C, the supernatant was removed, and cells were resuspended in ice-cold cytoplasmic extraction buffer supplemented with HALT protease/phosphatase inhibitor cocktail (no. 1861281, Fisher) and incubated with rotation for 10 min at 4°C. Cells were then centrifuged at 500*g* for 5 min at 4°C, and the supernatant was discarded. The pellet was resuspended in ice-cold membrane extraction buffer (MEB) containing HALT, briefly vortexed for 5 s, and incubated with rotation for 20 min at 4°C. Samples were pelleted at 3000*g* for 5 min at 4°C, the supernatant was removed, and fresh ice-cold MEB containing HALT was added. Samples were vortexed for 15 s and incubated with rotation for 30 min at 4°C. Following centrifugation at 5000*g* for 5 min at 4°C, the supernatant was discarded, and the pellet was resuspended in room temperature nuclear extraction buffer supplemented with 5 mM CaCl_2_ and micrococcal nuclease (3 U/μl). Samples were vortexed briefly (15 s) and incubated for 15 min at 37°C. After incubation, samples were vortexed again for 15 s and centrifuged at 16,000*g* for 5 min at 4°C. The chromatin-bound nuclear extract in the supernatant was collected. Equal volumes of these extracts were mixed with Laemmli buffer, separated by SDS–polyacrylamide gel electrophoresis (PAGE), and analyzed by immunoblotting using an SPHK2-specific antibody. Antibodies against histone H3 and glyceraldehyde-3-phosphate dehydrogenase were used as markers for chromatin and cytosolic fractions, respectively.

### Cell metabolic analyses

Mitochondrial respiration and ATP production rates were measured using the Seahorse XF Cell Mito Stress Test Kit (no. 103015-100, Agilent Technologies) and the Seahorse XF Real-Time ATP Rate Assay Kit (no. 103592-100, Agilent Technologies) with the Seahorse XFe24 Analyzer (Agilent Technologies). Data were normalized to protein content per well, as determined by a BCA Protein Assay Kit, and analyzed using the report generators provided by Agilent Technologies.

### Immunocytochemistry

Mature primary adipocytes cultured on Millicell EZ Slides (no. PEZGS0816, Sigma-Aldrich) were preincubated, where indicated, with 3.8 μM BODIPY 493/503 (no. D3922, Sigma-Aldrich) diluted in maintenance medium for 15 min at 37°C and 5% CO_2_ for identification of lipid droplets. Adipocytes, including those for BODIPY 493/503 experiments, were washed with ice-cold PBS and fixed with 4% (w/v) paraformaldehyde (no. 043368.9M, Fisher) in PBS (no. 10010023, Fisher) for 15 min before permeabilization with 0.1% Triton X-100 (no. AAA16046AE, Fisher) in PBS for 3 min, all at room temperature. Adipocytes were incubated with blocking buffer containing 5% fetal bovine serum and 1% BSA in PBS for 45 min at room temperature and subsequently incubated with primary antibodies diluted in blocking buffer overnight at 4°C in a dark/humidified chamber. Cells were then washed three times in ice-cold PBS for 10 min each and subsequently incubated with fluorescent secondary antibodies for 1 hour at room temperature in a dark/humidified chamber. Adipocytes were then washed three times in ice-cold PBS for 10 min each and mounted with VECTASHIELD Vibrance antifade mounting medium containing DAPI (no. H-1800, Vector Laboratories).

### Confocal immunofluorescence microscopy

An inverted Zeiss LSM880 confocal laser scanning microscope equipped with a 63× PlanApo oil immersion lens (numerical aperture, 1.4) was used to obtain micrographs of in vitro and in vivo immunofluorescence staining. For each experiment, the laser intensity remained constant, with the confocal pinhole set to 1 Airy unit for an emission wavelength of 520 nm, and the confocal pinhole for the other captured emission wavelengths (channels) within that experiment set to the same size to achieve optical sectioning. DAPI fluorescence was excited using a 405-nm laser diode, Alexa Fluor 488 and BODIPY 493/503 excited with a 488-nm argon-ion laser, and Alexa Fluor 555 excited using a 561-nm diode-pumped solid-state laser. DAPI, Alexa Fluor 488 and BODIPY 493/503, and Alexa Fluor 555 emissions were detected in the wavelengths of 410 to 470 nm, 490 to 550 nm, and 560 to 625 nm, respectively. The 16-bit micrographs were captured using Zen Black (Carl Zeiss Microscopy) and quantified using ImageJ.

For in vitro quantification of lipid droplets, the 16-bit images of BODIPY 493/503 were equally threshold, and their size and number were calculated automatically by particle analysis after selecting a region of interest around each cell. The 16-bit images of Alexa Fluor 555 emissions detecting UCP1 were equally threshold and a region of interest drawn around each cell to calculate % area stained per cell. For in vivo quantification, 16-bit images of Alexa Fluor 488 and 555 emissions were equally threshold to detect UCP1 or SPHK2 and PPARγ, respectively. % Area per field (63×) of UCP1 and PPARγ or % area of SPHK2 covering the nucleus by selecting a region of interest around each DAPI-positive nucleus was measured.

### Quantification of sphingolipids by mass spectrometry

Sphingolipids were quantified by LC-ESI-MS/MS (API 5500 QTRAP; ABSciex) from 10 μl of whole blood, 10 μl of plasma, 10 mg of liver, or 5 mg of adipose. Homogenized tissue, blood, or plasma samples were added to 13 mm–by–100 mm borosilicate tubes (no. 53283-800, VWR) with phenolic-lined polytetrafluoroethylene (PTFE)–Faced/14B white rubber liners (no. 60826-304, VWR) containing ice-cold HPLC-grade 3 ml of CH_3_OH, 100 μl of ddH_2_O (no. JT9831-3, VWR), and 10 μl of in-house internal standard cocktail. Internal standards contained 250 pmol of each species of unnaturally occurring 17-carbon chain length sphingoid bases [C17-sphingosine (no. 860640P, Avanti); C17-dihydrosphingosine (no. 860654P, Avanti); C17-S1P (no. 860641P, Avanti); C17-dihydrosphingosine-1-phosphate (no. 860655P, Avanti)] and 12-carbon chain length fatty acid *N*-acyl sphingolipids analogs [C12-Ceramide (no. 860512P, Avanti); C12-sphingomyelin (no. 860583P, Avanti); C12-glucosylceramide (no. 860543P, Avanti); and C12-Lactosyl(ß)Ceramide (no. 860545P, Avanti)] in a total volume of 10 μl (7:2:1, EtOH:MeOH:H_2_O).

Each sample was thoroughly sonicated for 30 s at room temperature, and, then, 1.5 ml of CHCl_3_ (no. CX1054-1, Sigma-Aldrich) was added, vortexed for 5 s, and sonicated for a further 30 s before the single-phase mixture was incubated overnight at 48°C. Extracts were centrifuged at 4200*g* for 10 min at 4°C, the supernatant transferred to fresh glass tubes (no. 40-4000-003, Colonial Scientific), and lipid extracts were reduced to dryness using a speed vac before reconstituting in 600 μl of the starting mobile phase solvent (MeOH:H_2_O, 1:1). Reconstituted lipids were sonicated for 30 s at room temperature and centrifuged at 4200*g* for 10 min at 4°C, and the supernatant was transferred to autoinjector vials (no. 10781-890A, VWR) with PTFE/silicone caps (no. 89239-020A, VWR) for LC-ESI-MS/MS analysis. Sphingolipids were separated by reverse-phase HPLC using a Supelco 2.1 (inside diameter) × 50-mm Ascentis Express C18 (2.7 μm) column (no. 53822-U, Sigma-Aldrich) with a binary solvent system at a flow rate of 0.5 ml/min with a column oven set at 35°C. Before sample injection, the column was first equilibrated for 30 s with a solvent mixture of 95% mobile phase A1 (CH_3_OH/H_2_O/HCOOH, 58/41/1, v/v/v, with 5 mM ammonium formate) and 5% mobile phase B1 (CH_3_OH/HCOOH, 99/1, v/v, with 5 mM ammonium formate). After sample injection (40 μl), the A1/B1 ratio was maintained at 95/5 for 135 s, followed by a linear gradient to 100% B1 over 90 s, and then held at 100% B1 for 330 s, followed by a 30-s gradient return to 95/5 A1/B1. After each run, the column was again equilibrated with 95:5 A1/B1 for 30 s. The Shimadzu Nexera LC-30 AD binary pump system coupled to a SIL-30 AC autoinjector and DGU20A_5R_ degasser (HPLC component) was coupled to an AB Sciex 5500 quadrupole/linear ion trap (QTrap; SCIEX) operating in triple quadrupole mode. Q1 and Q3 were set to pass molecularly distinctive precursor and product ions (or a scan across multiple mass/charge ratio in Q1 or Q3), using N_2_ to collisionally induce dissociations in Q2 (which was offset from Q1 by 30 to 120 eV). The ion source temperature was set at 300°C. Peaks were integrated against internal standards using Analyst (ABSciex).

### Western blotting

Equal weights of frozen tissue were homogenized in radioimmunoprecipitation assay (RIPA) buffer containing 50 mM tris-HCl (pH 7.4), 1 mM EDTA, 150 mM sodium chloride, 0.1% SDS, 1% Triton X-100, and 0.5% sodium deoxycholate, with HALT protease/phosphatase inhibitor cocktail (no. 1861281, Fisher). Tissues were placed in 1.5-ml Eppendorf tubes with 300 μl of RIPA buffer, homogenized with a pestle (no. K749521-1590, Thermo Fisher Scientific), and left on ice for 1 hour. Homogenates were centrifuged at 3000*g* for 5 min at 4°C, and the phase beneath the fat cake was placed in a fresh 1.5-ml tube and centrifuged again at 12,000*g* for 15 min at 4°C. Protein concentration was measured in the remaining supernatant using the Pierce BCA Protein Assay Kit (no. 23227, Fisher) and diluted with Laemmli buffer. Lysates containing equal amounts of protein were incubated at 1200 rpm for 1 hour at 55°C, separated by 10% SDS-PAGE, and transferred to nitrocellulose (no. 1620112, Bio-Rad) using the PierceG2 Fast Blotter (no. 62287, Fisher). Membranes were blocked for 1 hour in Tris-Buffered Saline with Tween-20 (TBST) containing 1.4 M NaCl, 27 mM KCl, 250 mM tris base (pH 7.4), and 0.1% Tween 20 (no. BP337, Fisher) with 5% BSA (no. A7906, Sigma-Aldrich) before incubating overnight with agitation at 4°C with primary antibodies diluted in TBST containing 5% BSA. Membranes were then washed three times in TBST for 10 min each before being incubated with agitation at room temperature with peroxidase-conjugated secondary antibodies diluted in TBST containing 5% BSA. Membranes were then washed a further three times in TBST for 10 min each. Protein bands were visualized using chemiluminescent substrate (no. 34578, Fisher), imaged in the linear range using an Azure Imager C600 (Azure Biosystems Inc.), and analyzed by ImageJ for densitometric quantification normalized to loading controls.

### RNA extraction and qPCR

RNA was extracted from equal weights of frozen tissue or cell densities using TRIzol (no. 15596026, Fisher). Cells were directly scraped into 1 ml of TRIzol, and tissues were homogenized in 1 ml of TRIzol for 1 min until visibly homogenized using a Tissue Tearor (no. 985370-07, BioSpec products). The cells or homogenates were placed at room temperature for 10 min, centrifuged at 12,000*g* for 10 min at 4°C, and, on ice, the TRIzol phase below the insoluble lipid phase was transferred to a new tube. Chloroform (200 μl; no. C2432, Sigma-Aldrich) was added, and the samples vigorously shaken and vortexed for 15 s each. Samples were left at room temperature for 3 min before being centrifuged at 12,000*g* for 15 min at 4°C. On ice, the aqueous phase was kept, and 1 μl of glycogen (no. 10901393001, Sigma-Aldrich) and 500 μl of isopropanol (no. A416, Fisher) were added and then inverted 15 times. After incubating for 10 min at room temperature, the mixture was centrifuged at 12,000*g* for 10 min followed by 22,000*g* for 1 min at 4°C to sequentially remove all supernatant. On ice, 1 ml of 75% ethanol (no. BP2818, Fisher) was added to the pellet, inverted 15 times, and kept overnight at −20°C. The sample was then centrifuged at 7500*g* for 5 min followed by 22,000*g* for 1 min at 4°C to sequentially remove all ethanol before being left to air dry on ice for 30 min. RNA was resuspended in 20 to 40 μl of ddH_2_O, DNase I–treated (no. E1011, Zymo Research), and concentration was measured using a NanoDrop 2000 (no. ND2000, Fisher). Two micrograms was converted to cDNA with the High-Capacity cDNA Reverse Transcription Kit (no. 4368814, Fisher), and mRNA expression was determined by mixing 20 ng of cDNA, SYBR (no. 4367659, Fisher), and primers specified in table S1. SYBR products were measured using a CFX Opus 96 Real-Time PCR Detection System (no. 12011319, Bio-Rad), and relative fold changes in gene expression were determined by 2^−∆∆Ct^ normalized to *PPIA*.

### Bulk RNA-seq

Total RNA was extracted from gWAT, sWAT, and BAT after 18 weeks chow or WD feeding using the protocol described above. RNA-seq was performed by the Genomics, Epigenomics and Sequencing Core (GES Core) at the University of Cincinnati. Quality [RNA integrity (RIN) 8.5 to 9.5], and concentrations of RNA were measured using a 2100 Bioanalyzer (no. G2939A, Agilent). Poly(A) RNA was isolated from 1 μg of total RNA using the NEBNext Poly(A) mRNA Magnetic Isolation Module (no. E7490S/L, New England Biolabs) and enriched with a SMARTer Apollo Library Prep System (no. 640078, Takara). The library was prepared using a dUTP-based stranded NEBNext Ultra II Directional RNA Library Prep Kit for Illumina (no. E7760S, New England Biolabs), indexed, and amplified with 8 PCR cycles. Uniquely barcoded libraries were then analyzed with the Bioanalyzer, proportionally pooled, and sequenced with the single read 1 × 85–base pair setting using an Illumina 2000 Sequencer (Illumina, RRID:SCR_023614) that generated ∼25 million pass filter reads per sample.

Fastq files were subsequently generated with Illumina BaseSpace, transferred to VCU High Performance Research Computing Core (HPRC) Fenn cluster, and analyzed by VCU Massey Comprehensive Cancer Center Bioinformatics Shared Resource. Raw fastq files were checked for quality using FastQC and collated using MultiQC for review. Adapters were trimmed using CutAdapt, and paired end 100–base pair reads were aligned using STAR (Aligner) version 2.7.9a to the mouse GRCm39/mm39 mouse primary assembly genome (https://ftp.ensembl.org/pub/release-108/fasta/mus_musculus/dna/) using default parameters (*90*).

Raw counts were generated using the FeatureCounts tool from the SourceForge Subread package and subsequently counted genes using the ensembl gtf file for *Mus musculus*, GRCm39 assembly, version release 108 (https://ftp.ensembl.org/pub/release-110/gtf/mus_musculus/). Raw counts were then analyzed using EdgeR package to generate TMM normalization data and perform differential expression between comparison groups using a standard pipeline (*91*). Low expressing genes were filtered, and the quasi-likelihood *F* test was used to test differential expression between groups. The *P* value was adjusted using the Benjamini-Hochberg adjustment for a false discovery rate (FDR). A significant differentially expressed gene was defined as FDR < 0.05. The *P* values and differential expressions (fold change) were used for downstream analysis.

Gene signatures of gWAT, sWAT, and BAT from Sphk2^∆Adipo^ male mice fed WD were compared with those of littermate controls, visualized as volcano plots, and analyzed using Database for Annotation, Visualization, and Integrated Discovery (DAVID) software [National Institutes of Health (NIH), RRID:SCR_001881] (*92*, *93*). GO functional enrichment and KEGG were used to perform GSEA for “molecular functions” and “biological processes” using significantly differentially expressed genes (*P* ≤ 0.01, logFC of ±1). Subsequent analysis was performed using IPA (QIAGEN, RRID:SCR_008653) (*P* ≤ 0.01, logFC of ±1) to compare gene signatures of sWAT from WD-fed WT and Sphk2^∆Adipo^ littermates mice.

### Cleavage under targets and tagmentation

Single nuclei were isolated from WT and *Sphk2*^∆Adipo^ male mice after 18 weeks WD feeding using the Minute Single Nuclei and Cytosol Isolation Kit (no. AN-029, Invent Biotechnologies). Snap-frozen sWAT (160 mg) was finely minced on ice, transferred to 1.5-ml tubes containing 200 μl of N/C extraction buffer with HALT protease/phosphatase inhibitors, and homogenized using the provided pestle for 5 min. A further 500 μl of N/C extraction buffer with HALT protease/phosphatase inhibitors was added, homogenized for another 2 min, transferred to prechilled filter cartridges, and incubated at −20°C for 20 min. The cartridge was then centrifuged at 13,000*g* for 30 s at 4°C, and the pelleted flowthrough was resuspended by pipetting 20 times before transferring to a fresh 1.5-ml tube. The sample was pelleted at 1200*g* for 5 min at 4°C, the supernatant was removed, the walls of the tube were thoroughly dried, and residual lipid was removed using a cotton swab. The nuclei pellet was resuspended in 105 μl of ice-cold PBS containing HALT protease/phosphatase inhibitors (without EDTA) and 5% BSA by gently pipetting 30 times. The single-nucleus suspension (5 μl) was used for Trypan blue (0.4%) (no. 15250-061, Fisher) staining to count 5000 nuclei for each sample that was transferred to a fresh 1.5-ml tube to be used as the input for the Hyperactive Universal CUT&Tag Assay Kit for Illumina Pro (no. TD904, Vazyme).

For each sample, 10 μl of ConA Beads Pro were thoroughly resuspended in 100 μl of binding buffer, beads separated for 2 min using a Dynal Bead Separator, resuspended again in 100 μl of binding buffer, magnetically separated again for 2 min, and lastly resuspended in 10 μl of binding buffer then left on ice. The remaining 100 μl of single-nucleus suspensions were centrifuged at 600*g* for 5 min at 4°C, and supernatant was removed and gently but thoroughly resuspended in 100 μl of ice-cold NE Buffer before incubating on ice for 10 min. The samples were again centrifuged at 600*g* for 5 min at 4°C; the supernatants were removed; nuclei were resuspended in 100 μl wash buffer, transferred to the 1.5-ml tube containing activated ConA Beads Pro before being gently mixed by flicking five times, and incubated for 10 min at room temperature. The samples were then centrifuged 50*g* for 30 s at 4°C; nuclei-bead complexes were magnetically separated for 2 min; supernatant was removed; and complexes were resuspended in 50 μl of prechilled antibody buffer containing anti-H3K27ac, anti-H3K9ac, or IgG isotype control with overnight rotation at 4°C. Secondary antibodies were pre-prepared in a fresh 1.5-ml tube by diluting anti-rabbit IgG (H+L) in Dig-wash buffer. Nuclei-bead complexes were then magnetically separated for 2 min, supernatant was discarded, and secondary antibody was added and mixed by pipetting five times before incubating with rotation for 1 hour at 4°C. The samples were then centrifuged 50*g* for 30 s at 4°C and magnetically separated for 2 min, and the nuclei-bead complexes were resuspended in 200 μl of Dig-wash buffer. The samples were then washed with 200 μl of Dig-wash buffer a further two times and magnetically separated for 2 min, supernatant was removed, and 100 μl of Dig-300 buffer containing 0.04 μM pA/G-TnP Pro was added. The sample was gently but thoroughly mixed by pipetting five times and incubated for 1 hour at room temperature. The samples were then centrifuged 50*g* for 30 s at 4°C and magnetically separated for 2 min, supernatant was removed, and nuclei-bead complexes were resuspended in 200 μl of Dig-300 buffer a total of three times. After the final magnetic separation, supernatant was removed, and 50 μl of Dig-300 buffer containing 1× TruePrep Tagment Buffer L was added, mixed by pipetting five times, and incubated for 1 hour at 37°C. Following incubation, 0.05 pg of *Escherichia coli* λDNA spike-in diluted in 10% SDS was added (final volume added, 2 μl), mixed by pipetting five times, and incubated for 10 min at 55°C. The samples were then centrifuged 50*g* for 30 s at 4°C and magnetically separated for 2 min, and supernatant was transferred to a fresh 1.5-ml tube on ice. Resuspended DNA Extract Beads Pro (25 μl) were magnetically separated for 2 min, supernatant was discarded, 200 μl of 1× B&W buffer was added and magnetically separated again, supernatant was discarded again, and beads were resuspended in 50 μl of 2× B&W buffer. Activated DNA Extract Beads Pro were added to the transposase-treated nuclei-bead complexes, mixed by pipetting five times, and rotated for 1 hour at room temperature. Samples were then magnetically separated for 3 min, supernatant was removed and washed twice with 200 μl of 1× B&W buffer for 30 s each, and beads were left to air-dry for 5 min before being resuspended in 15 μl of ddH_2_O.

Libraries were amplified by combining 15 μl of samples with a unique combination of 5 μl of N5XX and N7XX dual-index adapters (no. TD202, Vazyme) and 25 μl of 2× CAM before performing a PCR reaction using the following setup: 72°C for 3 min, 95°C for 3 min, 15 cycles of 98°C for 10 s and then 60°C for 5 s, 72°C for 1 min, and held at 4°C. The PCR products were purified by adding 100 μl of resuspended VAHTS DNA Clean Beads (no. N411, Vazyme), mixed by pipetting 10 times, and incubated for 5 min at room temperature. Complexes were magnetically separated for 5 min, supernatant was discarded, beads were rinsed twice with 200 μl of 80% HPLC-grade ethanol for 30 s each and left to air-dry for 5 min, all while remaining on the magnetic rack. Once dry, DNA was eluted by adding 22 μl of ddH_2_O, mixed by pipetting 10 times, incubated for 5 min at room temperature, and magnetically separated for 5 min, and supernatant was collected as sample for sequencing.

The sequencing was performed by the GES Core at the University of Cincinnati. First, quality and concentration of the DNA libraries were assessed by a high-sensitivity dsDNA kit (no. 5067-4626, Agilent) run on a 2100 Bioanalyzer (no. G2939A, Agilent), yielding the expected quality and fragment lengths (∼200 to 1000 base pairs). Each library (0.5 nM) was then equal-volume pooled and sequenced to generate a few million reads. Based on the number of reads, the input from each library was adjusted to account for differences in total reads generated. Adjusting the volume to ensure that an equal number of reads from each library were sequenced, the final sequencing was performed using an Illumina NextSeq 2000 Sequencer (Illumina, RRID:SCR_023614) with PE 2 × 61–base pair settings. Between 35 and 40 million paired-end reads per sample were generated, processed, and analyzed following the protocol described in (*94*) and the accompanying tutorial (*95*). Briefly, FASTQ reads were aligned using Bowtie2 with parameters --local --very-sensitive --no-mixed --no-discordant --phred33 -I 10 -X 700 to the mouse mm39 (GRCm39) version 30 reference genome. To account for spike-in calibration, reads were also aligned to the *E. coli* genome using Bowtie2 with the --end-to-end mode and the same sensitivity and filtering parameters. Peak calling was performed using the SEACR (Sparse Enrichment Analysis for CUT&RUN) package (*94*). A unified peak-by-sample matrix was constructed by merging peaks identified in each individual sample. Fragment counts per peak were quantified, normalized for sequencing depth, and analyzed for differential enrichment using DESeq2 (*96*). Individual peak regions were visualized with Integrative Genomics Viewer 2.19.1, and GSEA analysis was performed with DAVID and GO biological processes using significantly differentially enriched regions (*P* ≤ 0.05, logFC of ±0.37) for WT or Sphk2^∆Adipo^ versus IgG controls and for WT versus *Sphk2*^∆Adipo^ unless otherwise indicated.

### HDAC and HDAC3 activities

Total HDAC activity was measured in sWAT nuclear extracts using the HDAC fluorometric cellular activity assay kit (no. BML-AK503-0001, Enzo Life Sciences). Fluorescence signals were converted to absolute values using a standard curve generated with a deacetylated standard and corrected for background activity from lysis buffer controls.

HDAC3 activity was measured as previously described with some modifications (*97*). Briefly, 80 to 100 mg of adipose tissue was homogenized in HDAC cell lysis buffer (no. BML-KI346-0020, Enzo Life Sciences) and incubated overnight at 4°C with 1 μg of HDAC3 antibody (no. 85057S, Cell Signaling Technology) or 1 μg of normal rabbit IgG (no. sc-2763, Santa Cruz Biotechnology) as a control. Immune complexes were captured with Protein A/G Plus-Agarose (no. sc-2003, Santa Cruz Biotechnology) for 4 hours at 4°C with rotation. Immunoprecipitates were collected by centrifugation (1000*g*, 5 min, 4°C) and washed four times with PBS. Pellets were resuspended in HDAC assay buffer, and aliquots were used for activity measurements. Fluorescence signals were quantified against a deacetylated standard curve. HDAC3-specific activity was calculated by subtracting IgG control activity from HDAC3-immunoprecipitated activity for each sample.

### Statistical analyses

For each experiment, *N* denotes biological replicates, and *n* is the number of technical replicates. All data are expressed as means ± SEM. Statistical analysis of *P* values was determined using GraphPad Prism 10 software. Statistical significance was determined between two groups using unpaired two-tailed *t* test. For multiple comparisons, one-way or two-way analysis of variance test (ANOVA) was used for comparisons with one or two factors, respectively, followed by Holm-Šidák or Tukey’s post hoc tests described in the figure legends. For all experiments, the normality of the data from each group was first checked using the Shapiro-Wilk test. The designations for significance levels are *P ≤ 0.05, **P ≤ 0.01, ***P ≤ 0.001, and ****P ≤ 0.0001, which also apply to # comparisons.
