# Supplementary material for: Sphingosine kinase 2 regulates adipocyte browning and whole-body metabolism
Source: Sci Adv. 2026 Aug 21;12(34):eaec7066. doi: 10.1126/sciadv.aec7066 (PMC13496211; doi:10.1126/sciadv.aec7066)
Supplement: Supplementary file 1 [file sciadv.aec7066_sm.pdf]

Science Advances
AAAS

Supplementary Materials for

Sphingosine kinase 2 regulates adipocyte browning and whole-body metabolism

Ryan D. R. Brown *et al.*

Corresponding author: Christopher D. Green, christopher.d.green@vcuhealth.org;
Sarah Spiegel, sarah.spiegel@vcuhealth.org

Sci. Adv. **12**, eaec7066 (2026)
DOI: 10.1126/sciadv.aec7066

This PDF file includes:

Figs. S1 to S7
Table S1

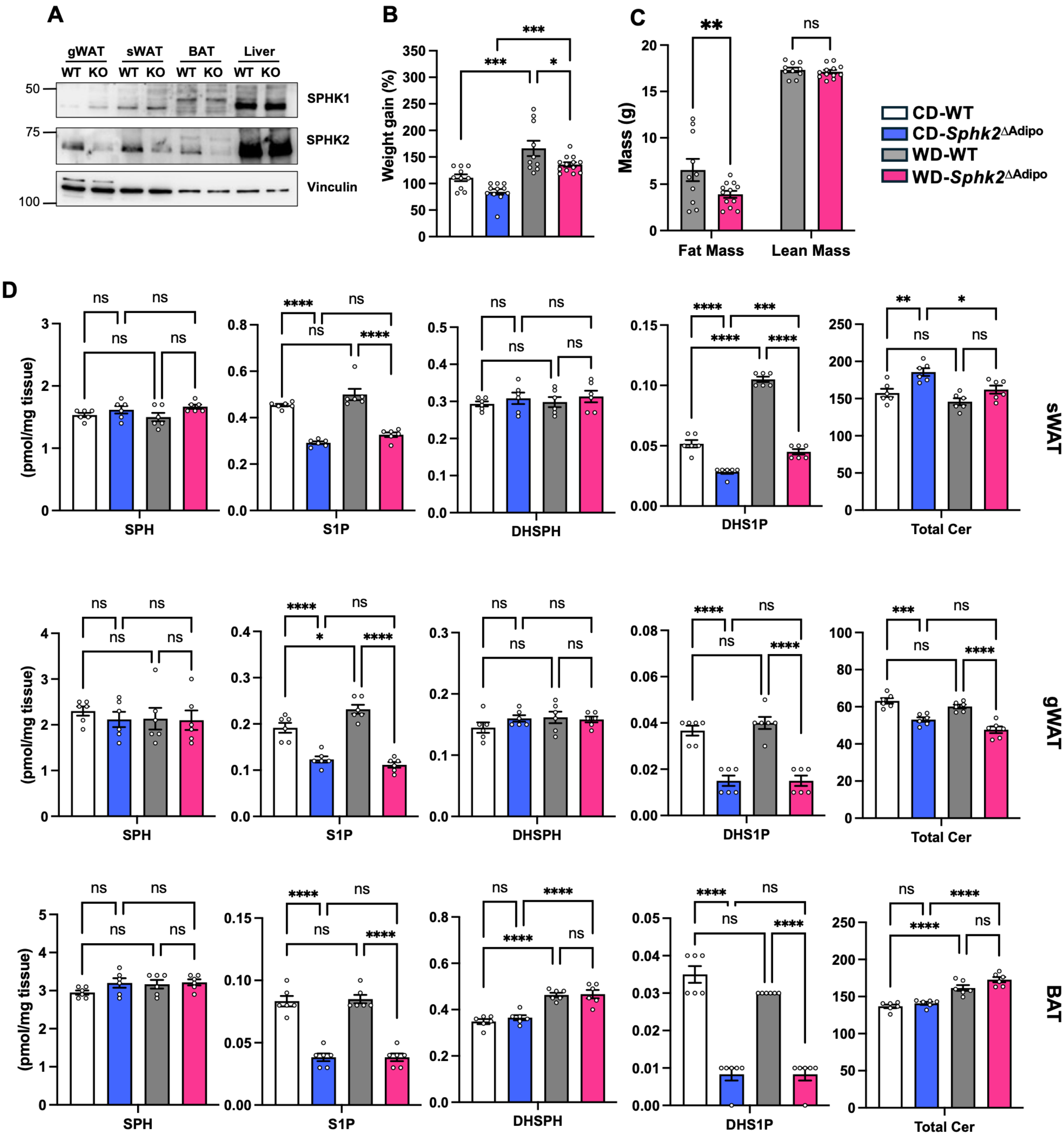

Figure S1. Adipose deletion of *Sphk2* in female mice reduces Western diet-induced increases in weight gain and S1P accumulation. (**A**) Western blots of SPHK1 and SPHK2 in gWAT, sWAT, BAT and liver from female wild-type (WT) and *Sphk2*$^{\Delta Adipo}$ (KO) mice (N=3). (**B-D**) WT and *Sphk2*$^{\Delta Adipo}$ female mice were fed chow or WD for 18 weeks as indicated. (**B**) weight gain, (**C**) body composition (N=10-13). (**D**) Sphingolipids were extracted from gWAT, sWAT and BAT and levels of sphingosine (SPH), dihydrosphingosine (DHSPH), sphingosine-1-phosphate (S1P), dihydrosphingosine-1-phosphate (DHS1P), and total ceramide were determined by LC-ESI-MS/MS (N=6). Data are mean ± SEM. (**B-D**) One-way analysis of variance test followed by Sidak's multiple comparison test.

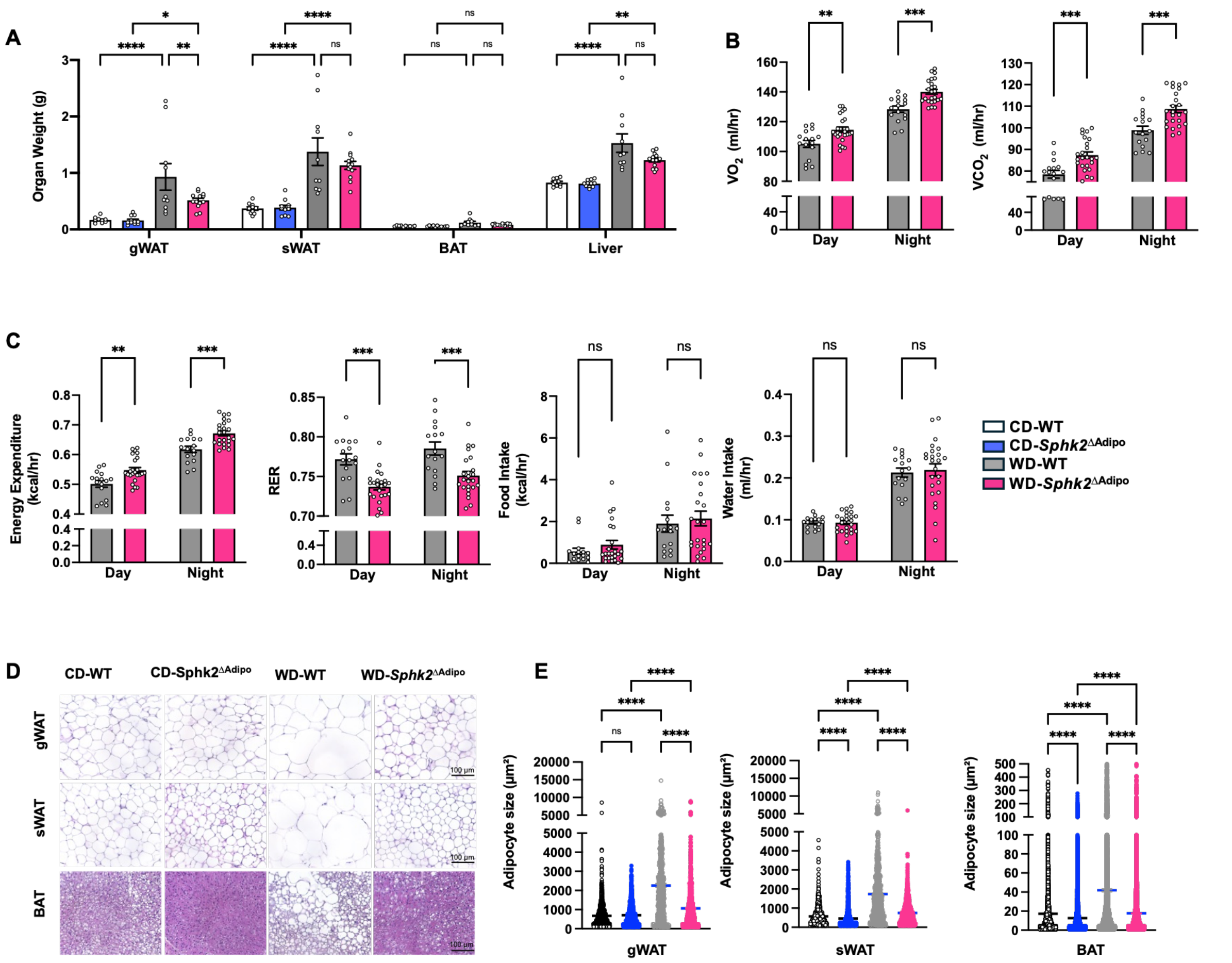

Figure S2. Adipocyte-specific ablation of *Sphk2* in obese female mice induces Adipose Tissue Remodeling and Increases Energy Expenditure. (**A-E**) Female *Sph*k2$^{\Delta Adipo}$ mice and WT littermates were fed either standard chow or a WD for 18 weeks. (**A**) Organ weights were measured (N=10-13). (**B,C**) Following 16 weeks of WD feeding female mice were individually housed in metabolic chambers for two days for acclimatization. Subsequently, measurements of water consumption, food intake, energy expenditure, oxygen consumption (VO2), carbon dioxide production (VCO2), and respiratory exchange ratio (RER) were recorded over the next four days and displayed as daily averages (N=16-24). (**D**) Representative images of hematoxylin-eosin (H&E) stained gWAT, sWAT and BAT are shown. (**E**) Adipocyte sizes were quantified in each depot (N=6). Data are mean ± SEM. (A) Two-way analysis of variance test followed by Tukey's multiple comparison test. (**B,C**)Two-way analysis of variance test followed by Sidak's multiple comparison test. (**E**) One-way analysis of variance test followed by Tukey's multiple comparison test.

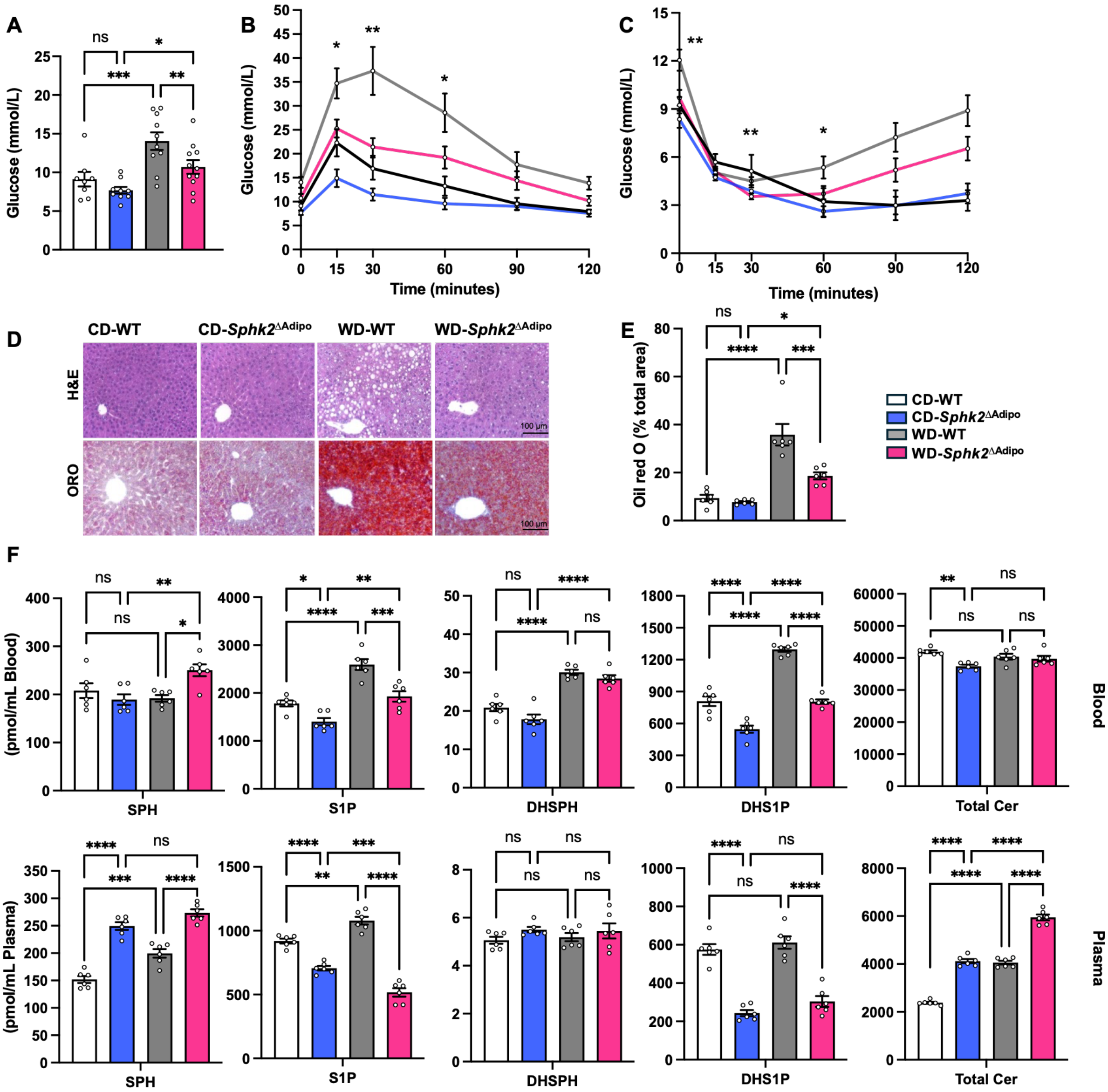

Figure S3. Improved glucose tolerance and reduced steatosis in WD fed *Sph*k2$^{\Delta\text{Adipo}}$ female mice. (A-F) *Sphk2*$^{\Delta\text{Adipo}}$ and WT female littermates were fed chow or WD for 15 (**B,C**) or 18 (**A,D,E,F**) weeks. (**A**) Blood glucose levels. (**B**) Glucose tolerance test. (**C**) Insulin tolerance test (N=8-11). (**D**) Representative images of hematoxylin-eosin (H&E) and oil-red O (ORO)-stained liver sections after 18 weeks chow or WD feeding (**E**). ORO staining was quantified as percent staining per total tissue surface area (N=6). (**F**) Sphingolipids were extracted from blood and plasma and levels of sphingosine (SPH), dihydrosphingosine (DHSPH), sphingosine-1-phosphate (S1P), dihydrosphingosine-1-phosphate (S1P), and total ceramide were determined by LC-ESI-MS/MS (N=6). Data are mean ± SEM. (**A,E,F**) One-way analysis of variance test followed by Tukey's multiple comparison test. (**B,C**) Unpaired two-tailed *t*-test at each time point comparing WD-WT to WD- *Sphk2*$^{\Delta Adipo}$.

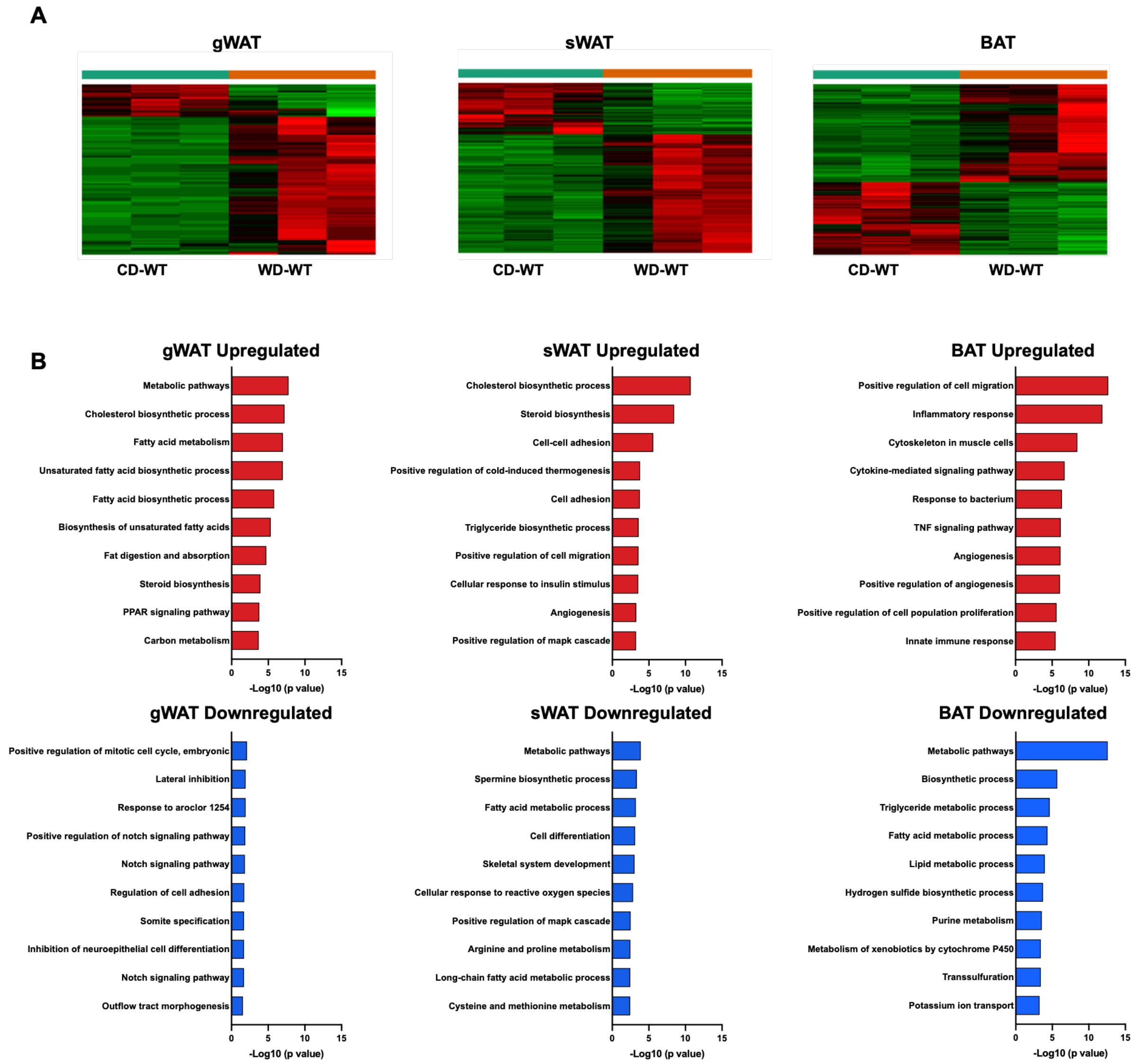

Figure S4. Effect of Western diet on the adipose tissue transcriptome. (**A, B**) WT male littermates were fed chow or WD for 18 weeks. (**A**) Heatmap expression matrix of differentially expressed genes in BAT, gWAT, and sWAT from WD-fed WT mice compared to chow-fed WT. (**B**) GO biological processes and KEGG pathway enrichment analysis of significant differentially expressed genes that are upregulated or downregulated in BAT, gWAT, and sWAT from WD-fed WT mice compared to CD-fed WT ($p \leq 0.01$, LogFC±1) (N=3).

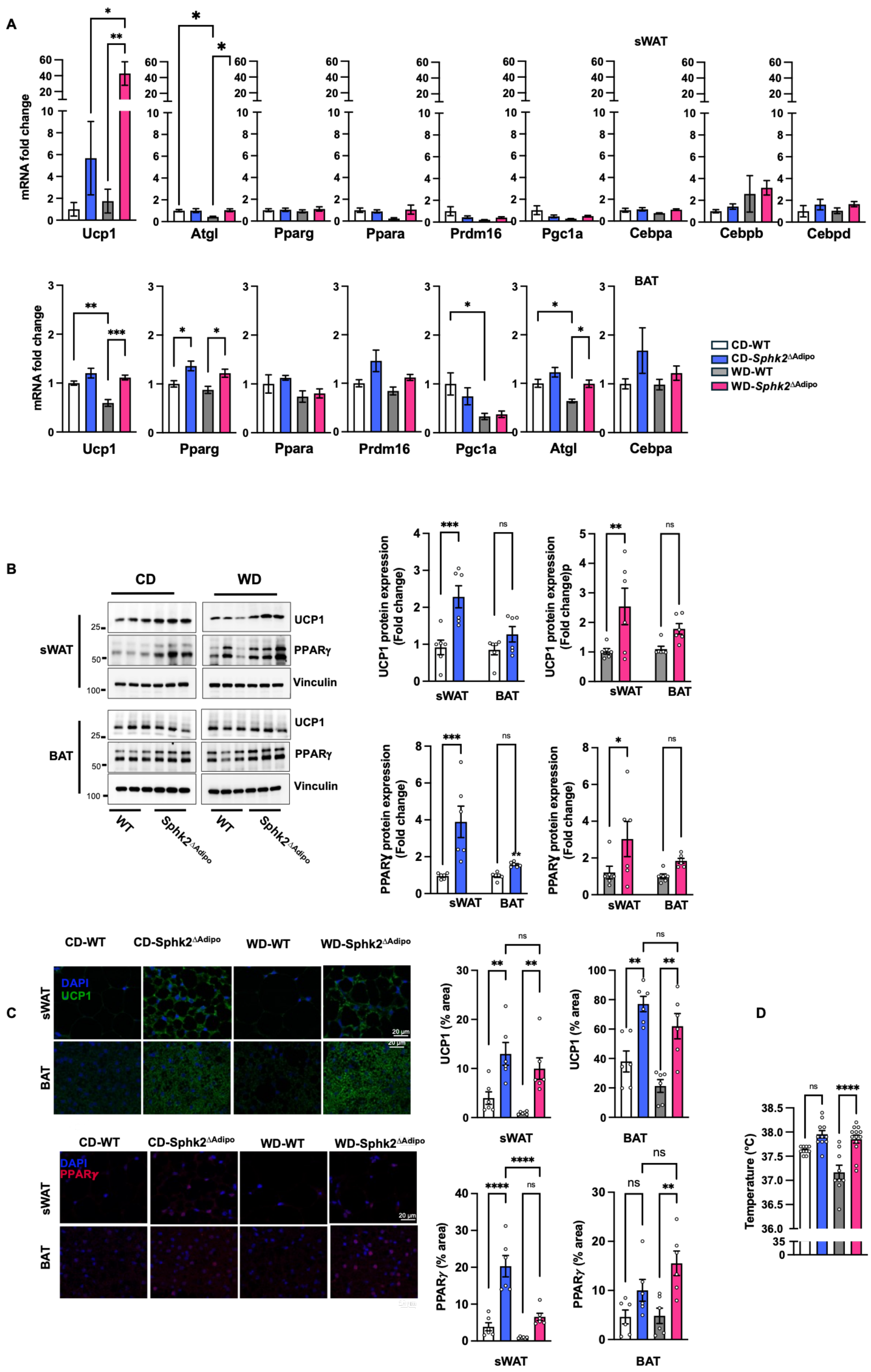

Figure S5. Sphk2 deletion in adipocytes of female mice stimulates expression of thermogenic genes. (**A-D**) *Sphk2*$^{\Delta\Delta Adipo}$ and WT female mice were fed chow or WD for 18 weeks. (**A**) Expression of thermogenesis and lipolysis genes in sWAT and in BAT, as indicated. (N=6). (**B**) Representative western blots of UCP1 and PPARγ in sWAT and BAT and densitometric analyses of blots. Vinculin was used as loading controls. (N=6). (**C**) Representative confocal micrographs of sWAT and BAT stained for UCP1 or PPARγ and counterstained with DAPI, with quantification shown as percent stained per field. (N=6). (**D**) Rectal temperatures. (N=10-13). (**A-D**) Data are mean ± SEM, one-way analysis of variance test followed by Tukey's multiple comparison test.

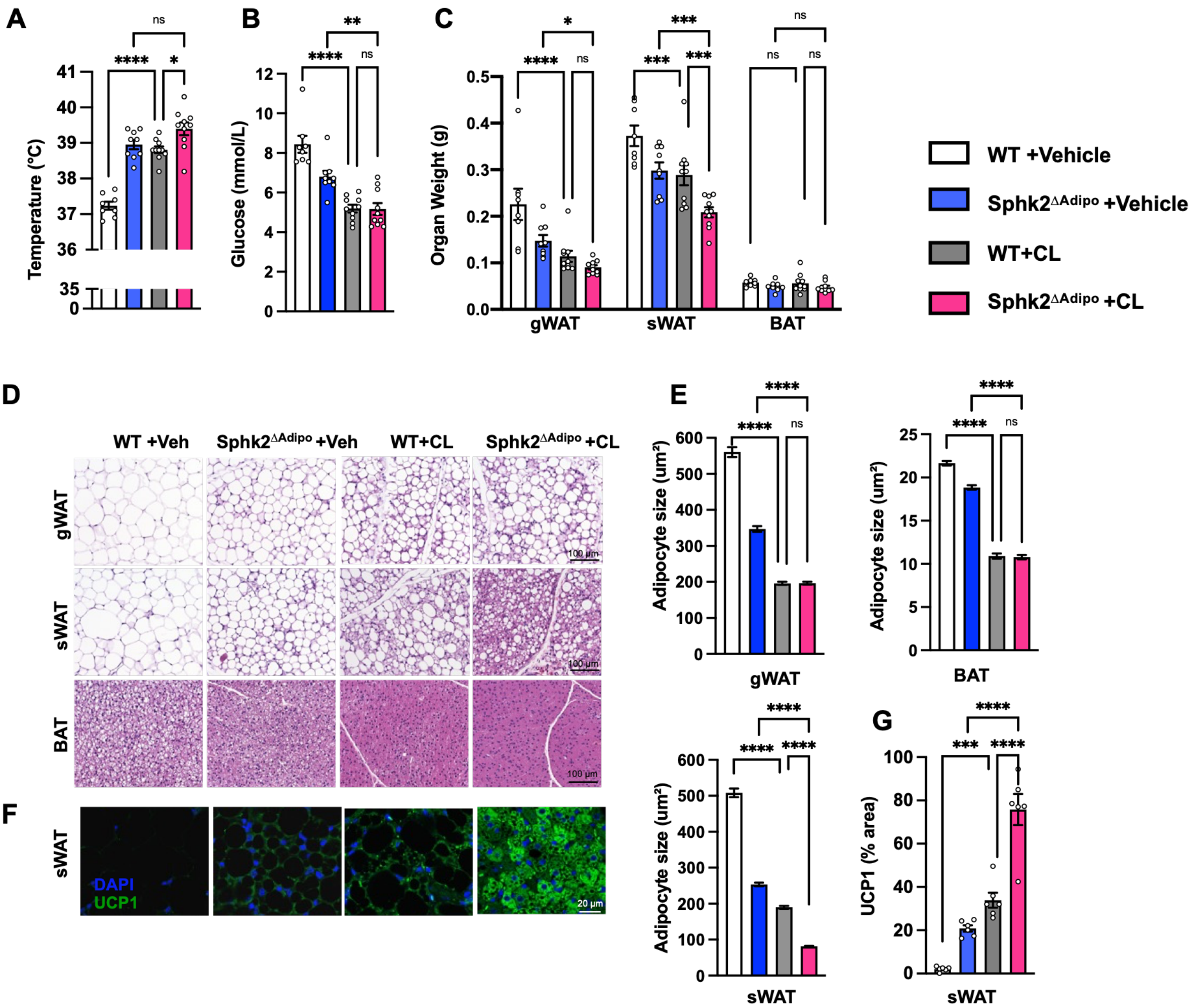

Figure S6. Adipocyte-selective depletion of *Sphk2* in female mice enhanced b-adrenergic agonist CL316,243 responsiveness. (**A-G**) *Sphk2*$^{\Delta Adipo}$ and WT female mice received daily injections of either vehicle or CL316243 for 7 days. (**A**) Rectal temperatures (N=8-10). (**B**) Fasted blood glucose levels. (**C**) Organ weights (N=8-10). (**D**) Representative images of H&E stained gWAT, sWAT, and BAT. (**E**) Adipocyte cell size (N=6). (**F**) Representative confocal micrographs of sWAT stained for UCP1 and counterstained with DAPI. (**G**) Percentage of UCP1 stained area per field (N=6). Data are mean ± SEM. (**A-C,E,G**). One-way analysis of variance test followed by Tukey's multiple comparison test.

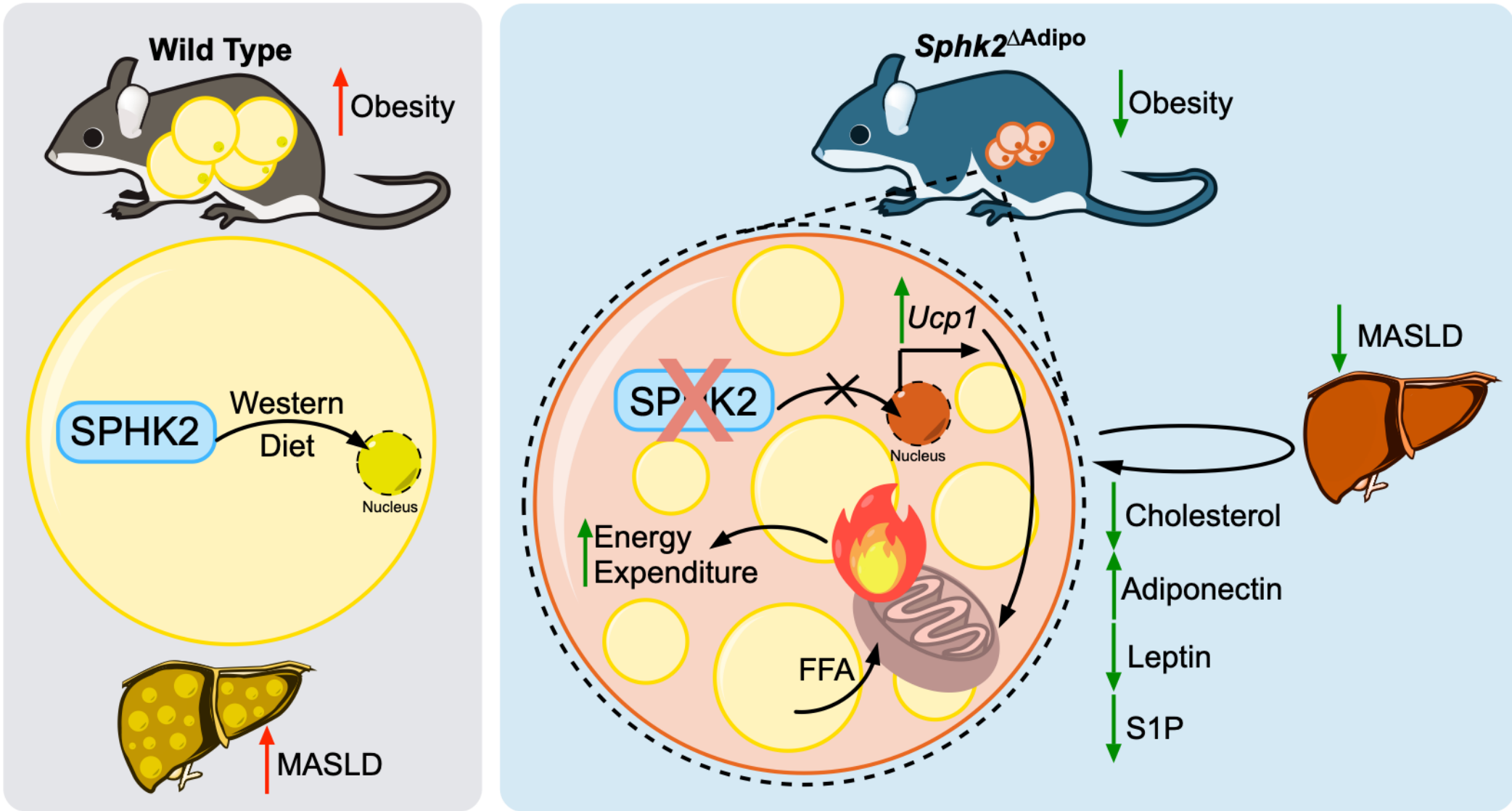

Figure S7. Schematic overview of the metabolic role of SPHK2 in adipocytes and its impact on obesity-associated metabolic dysfunction. Western diet feeding induces nuclear localization of SPHK2 in white adipocytes, where it represses the thermogenic gene program. Loss of SPHK2 in adipocytes increased UCP1 and energy expenditure associated with altered circulating factors, including decreased cholesterol, leptin, and S1P, and increased adiponectin, ultimately leading to protection against hepatic steatosis.

Supplementary Table 1. List of primers used in this study

Source	Gene	Polymerase	Forward 5'-3-	Reverse 5'-3-
Custom (IDT)	UCP1	SYBR	AGGCTTCCAGTACCATTAGGT	CTGAGTGAGGCAAAGCTGATT
BioRad	Sphk1	SYBR	NA	NA
BioRad	Sphk2	SYBR	NA	NA
Custom (IDT)	PPARγ	SYBR	TCGCTGATGCACTGCCTATG	GAGAGGTCCACAGAGCTGATT
Custom (IDT)	PPARα	SYBR	GCAGTGCCCTGAACATCGA	TCGCCGAAAGAAGCCCTTA
Custom (IDT)	PRDM16	SYBR	CCACCAGCGAGGACTTCAC	GGAGGACTCTCGTAGCTCGAA
Custom (IDT)	PGC1α	SYBR	AGACGGATTGCCCTCATTTGA	GGTCTTACCAATGGCAGGGTTT T
Custom (IDT)	CIDEA	SYBR	TCTGCAATCCCATGAATGTC	CAGTGATTTAAGAGACGCGG
BioRad	FGF21	SYBR	NA	NA
BioRad	TBX1	SYBR	NA	NA
Custom (IDT)	ATGL	SYBR	CAACGCCACTCACATCTACGG	GGACACCTCAATAATGTTGGCA C
BioRad	HSL	SYBR	NA	NA
BioRad	MGL	SYBR	NA	NA
BioRad	SIRT1	SYBR	NA	NA
Custom (IDT)	CD36	SYBR	CCTGGGAGTTGGCGAGAAA	CGATCACAGCCCATTCTCCT
Custom (IDT)	FABP4	SYBR	AAGGTGAAGAGCATCATAACC CT	TCACGCCTTTCATAACACATTC C
Custom (IDT)	SREBP1c	SYBR	CCACTAGAGGTCGGCATGGT	TCCCTTGAGGACCTTTGTCATT
Custom (IDT)	SREBP2	SYBR	GTTGACGCAGACAGCCAATG	CCCTTACTGGCACTTGAAGG
Custom (IDT)	HMGCoA R	SYBR	CTTGTGGAATGCCTTGTGATT	AGCCGAAGCAGCACATGAT
Custom (IDT)	FASN	SYBR	CTTCTGATCAGTGGCCTCCTC	GGATTCGGGAATACAAGTGGC
BioRad	ACACA	SYBR	NA	NA
Custom (IDT)	CEBPα	SYBR	CGGGAACGCAACAACATCGC	TGTCCAGTTCACGGCTCAGC
Custom (IDT)	CEBPβ	SYBR	ACCTGGAGACGCAGCACAAG	CTGCTTGAACAAGTTCCGCAG
Custom (IDT)	CEBPδ	SYBR	CCATGGATCGGAATAGTGAC	TTCACCGCCATATTGTTCC
